# TGPred: efficient methods for predicting target genes of a transcription factor by integrating statistics, machine learning and optimization

**DOI:** 10.1093/nargab/lqad083

**Published:** 2023-09-13

**Authors:** Xuewei Cao, Ling Zhang, Md Khairul Islam, Mingxia Zhao, Cheng He, Kui Zhang, Sanzhen Liu, Qiuying Sha, Hairong Wei

**Affiliations:** Department of Mathematical Sciences, Michigan Technological University, Houghton, MI 49931, USA; Computational Science and Engineering Program, Michigan Technological University, Houghton, MI 49931, USA; College of Forest Resources and Environmental Science, Michigan Technological University, Houghton, MI 49931, USA; Computational Science and Engineering Program, Michigan Technological University, Houghton, MI 49931, USA; College of Forest Resources and Environmental Science, Michigan Technological University, Houghton, MI 49931, USA; Department of Plant Pathology, Kansas State University, Manhattan, KS 66506, USA; Department of Plant Pathology, Kansas State University, Manhattan, KS 66506, USA; Department of Mathematical Sciences, Michigan Technological University, Houghton, MI 49931, USA; Department of Plant Pathology, Kansas State University, Manhattan, KS 66506, USA; Department of Mathematical Sciences, Michigan Technological University, Houghton, MI 49931, USA; Department of Mathematical Sciences, Michigan Technological University, Houghton, MI 49931, USA; Computational Science and Engineering Program, Michigan Technological University, Houghton, MI 49931, USA; College of Forest Resources and Environmental Science, Michigan Technological University, Houghton, MI 49931, USA

## Abstract

Four statistical selection methods for inferring transcription factor (TF)–target gene (TG) pairs were developed by coupling mean squared error (MSE) or Huber loss function, with elastic net (ENET) or least absolute shrinkage and selection operator (Lasso) penalty. Two methods were also developed for inferring pathway gene regulatory networks (GRNs) by combining Huber or MSE loss function with a network (Net)-based penalty. To solve these regressions, we ameliorated an accelerated proximal gradient descent (APGD) algorithm to optimize parameter selection processes, resulting in an equally effective but much faster algorithm than the commonly used convex optimization solver. The synthetic data generated in a general setting was used to test four TF–TG identification methods, ENET-based methods performed better than Lasso-based methods. Synthetic data generated from two network settings was used to test Huber-Net and MSE-Net, which outperformed all other methods. The TF–TG identification methods were also tested with SND1 and *gl3* overexpression transcriptomic data, Huber-ENET and MSE-ENET outperformed all other methods when genome-wide predictions were performed. The TF–TG identification methods fill the gap of lacking a method for genome-wide TG prediction of a TF, and potential for validating ChIP/DAP-seq results, while the two Net-based methods are instrumental for predicting pathway GRNs.

## INTRODUCTION

Construction and delineation of transcriptional regulatory networks are essential for a systematic understanding of how various biological processes and complex traits are regulated and how plants grow and develop in response to environmental cues. Although biological experiments can be carried out to obtain gene regulatory relationships, they are labor-intensive and time-consuming, and can therefore only be used to infer a small number of true regulatory relationships. In the last two decades, the advent of high throughput technologies, including microarray and RNA-Seq, has made it easier to generate a terabyte of transcriptome data for inferring gene regulatory relationships and gen regulatory networks (GRNs). As the high-throughput data in public repositories increases exponentially, computational algorithms and tools that utilize high-throughput transcriptome data and ChIP/DAP-seq data provide an alternate approach to infer gene regulatory relationships and GRNs. However, the validity of this approach relies on the accuracy of the methods.

Initially, high-throughput transcriptome data sets were primarily generated from the single-celled organisms like bacteria and yeast, or the eukaryotic cell lines. These organisms and cell lines allowed for the generation of time-course microarray data with small time intervals (e.g. 5–10 min), which spurred the development of dynamic methods, including differential equations (1), finite state linear model (2), dynamic Bayesian (3), Boolean network (4), and stochastic networks (5) and ordinary differential equations (ODE) (6). These methods require time-course data sets with very small-time intervals for inferring gene regulatory relationships and GRNs due to the use of temporal variables. When microarray and RNA-seq were applied to multicellular organisms such as mammals and plants, acquisition of data from specific cells or tissues in a time series became challenging and time-consuming. As a result, most transcriptome data sets generated from multicellular organisms are static data, which are either in a non-time-course (e.g. treatments vs controls) or in a small time-course with very large time intervals (e.g. on a scale of many hours, days or weeks). This kind of data can be classified into static data because implementation of dynamic methods as mentioned above to this kind of data is inappropriate. Since it is not appropriate to analyze static data using dynamic methods, a number of static algorithms have been developed that do not rely on any temporal variables to simulate gene regulatory relationships. These methods includes ParCorA (7), Maximum Relevance/Minimum Redundancy Network (MRNET) (8), Mutual Information Based Relevance Networks (9), Trustful Inference of Gene Regulation using Stability Selection (TIGRESS) (10), Algorithm for the Reconstruction of Accurate Cellular Networks (ARACNE) (11), Context Likelihood of Relatedness (CLR) (12), Mutual Information 3 (MI3) (13), Probabilistic-Based Bayesian Network(14) and Random Forests (15). Recently, more methods have been developed for constructing multilayered hierarchical gene regulatory networks (ML-hGRNs), such as top-down (16,17), and bottom-up GGM algorithms (18), and BWERF(19), and GRNs that controls a pathway or a biological process, for instance, TGMI (20) and HB-PLS (21). In addition, the methods for constructing multiple joint GRNs using data from multiple tissues or conditions have been developed, for instance, JGL (22) and JRmGRN (23). Some recent studies (21,23) have shown that the integration of machine learning, statistics and optimization for inferring transcription factor (TF)-target gene (TG) relationships is promising and may open a new avenue for identifying regulatory relationships and constructing GRNs.

Plant researchers often produce a large number of transgenic lines in which a TF is up-/down-regulated, or use a transient expression system, for instance, CRISPR-CAS9 (24) or dTALE (25), to perturb a TF and then generate transcriptome data after the TF is overexpressed, suppressed or perturbed. Even with this kind of data, identification of the true target genes of the TF for further experimental studies is still a challenging task, even after a ChIP-seq or DAP-seq experiment of this TF is conducted. This is because ChIP-seq and DAP-seq experiments often yield hundred up to twenty thousand putative target genes. In addition, the ChIP/DAP-seq results show if a TF can bind to the promoters of candidate target genes but do not provide the information if the putative target genes are actually activated/suppressed by the TF. Therefore, in-silico methods that enable identification of the true target genes of a TF using the above-mentioned transcriptomic data is desperately demanding. The aforementioned dynamic and static network construction methods are often not specifically tailored for inferring the true TGs of a TF from large number of candidate TGs.

In this study, four statistical selection methods were developed to infer the potential TGs for a given TF by combining two loss functions, mean squared error (MSE) or Huber loss function (Huber), with two penalty functions, elastic net (ENET) or least absolute shrinkage and selection operator (Lasso). These four methods are referred to as Huber-ENET, Huber-Lassso, MSE-ENET and MSE-Lasso. The MSE and Huber loss functions were used to measure the errors between the predicted values and the observed values. Huber can more readily avoid the sensitivity of heavy-tailed errors or outliers than MSE. The penalty functions, ENET and Lasso contain the ${l_1}$ norm of the estimated effect sizes which can control the sparsity of the selected TGs. In addition, a network-based penalty (Net) was proposed, and combined with Huber or Lasso loss function to develop two Net-based methods, referred to as Huber-Net and MSE-Net, which can be used to identify pathway GRNs. Net penalty can incorporate prior annotated pathway or biological process information into the prediction (26).

To solve the regressions for the methods described above, an accelerated proximal gradient descent (APGD) algorithm was developed for the parameter optimization in all six methods. Our simulations showed that the APGD was equally effective but much faster than a commonly used method called convex optimization solver (CVX). To obtain stable selection results, we applied a stability selection method, the half-sample approach, which does not need to choose the optimal tuning parameters in selection methods. All the methods were tested using simulated data, and the four TF–TG identification methods were also tested with the real transcriptomic data from SND1 and *gl3* overexpression studies. In addition, the two Net methods were tested with the real transcriptome data of all metabolic pathway genes from the maize B73 line from public repository. Our study showed that the four TF–TG identification methods had higher efficacy in genome-wide prediction than the three comparison methods, CLR, MRNET and TIGRESS, implying that the methods can be used to validate TGs of a TF resulting from ChIP-seq or DAP-seq experiments, while the two Net-based methods can identify pathway GRNs.

## MATERIALS AND METHODS

### Simulated gene expression data

The simulated data were generated in three settings: (i) a general setting; (ii) two network settings: a hierarchical network setting and a Barabasi-Albert (BA) network setting. In the general setting, $p$ TGs were independent with each other and the first 50 TGs were regulated by a given TF (details in Supplemental Text S1). In the network settings, we simulated $p$ TGs with two biological network structures, the hierarchical network and Barabasi-Albert network. For the hierarchical network, there were 5 disjointed subnetworks and each of them consisted of 100 TGs. The subnetwork was constructed as previously described (26) (Supplementary Figure S1). For the Barabasi-Albert (BA) network, there were 50 subnetworks and each of them was a BA-based network comprising of 10 TGs (27). There were 45 TGs and 40 TGs that were regulated by a given TF for the hierarchical network and Barabasi-Albert network, respectively (details in Supplemental Text S2).

### *Populus trichocarpa* SND1 overexpression transcriptomic data and analysis

The poplar data used for simulation was from our previous study (16). The data can be retrieved from Gene Expression Omnibus (GEO) with accession number GSE49911. Briefly, the data was generated and then analyzed as following: Poplar protoplasts isolated from stem developing xylem were transfected with the plasmid vector harboring poplar SND1, a TF that is known to control lignocellulosic biosynthesis, under the control of 35S promoter, and were then harvested for RNA-seq at 7, 12 and 25 h. Three samples of transfected protoplasts (35S-SND1) and three control samples (control vector without SND1) at each time point were harvested. The raw read counts of all genes of each sample were used for identification of differentially expressed genes (DEGs) at each time point using the edgeR package(28). Meanwhile, the raw reads of all genes of each sample were normalized with trimmed mean of *M*-values (TMM), a scaling method contained in the edgeR package. The normalized data was used for real data simulation to validate the methods we developed in this study.

### Maize *gl3* overexpression transcriptomic data and analysis

Two transcriptional-activator like effectors (dTALes) that target two non-overlapping 16-bp regions of the *gl3* promoter for overexpression were constructed. The two regions targeted are located 5 and 48 bp upstream of the *gl3*’s transcription start site. The 14-day-old seedlings were used to test the dTALes-mediated induction of *gl3*. Three samples and three controls, upon being infected with Xv1601 bacteria carrying dTALes, dT1 or dT2, were harvested in a time-series with four time points: 18 and 24 h. Sequencing data were trimmed by Trimmomatic (version 0.38) (29) and trimmed reads were aligned to the maize B73 reference genome (B73Ref4) using STAR (2.7.3a) (30). Fragments per kilobase of transcript per million mapped reads FPKM values were generated with Cufflink package (31), and DEGs were identified with Cuffdiff package (32). FPKM data were used for simulation with *gl3* as a TF and all DEGs or all genomic genes as candidate target genes.

### Maize B73 transcriptomic data for validation of net-based methods

In total, the expression levels of 736 RNA-seq data of B73 were downloaded from NCBI Sequence Read Archive (SRA) repository. The accession numbers are shown in Table S1. The sequence reads were preprocessed as described for *gl3* data as described above. 2539 unique pathway genes were extracted from the Plant Metabolic Network (PMN) (33) and 23 lignin pathway genes as well as 23 transcription factors (TFs) that are known to regulate lignin pathway (34–38) were used for validating the two Net-based methods, Huber-Net and MSE-Net, with three network construction methods, CLR, MRNET and TIGRESS used as comparison.

### Rationale for methods

Consider that the expression levels of a TF $y$ and the expression levels of the TGs $x$ in the whole-genome fit a linear relationship of the following:


(1) \begin{equation*}{y_i} = {\beta _0} + x_i^T\beta + {\varepsilon _i},\;i = 1, \cdots ,n\end{equation*}

where $n$ is the number of samples, ${x_i} = {( {{x_{i1}}, \cdots ,{x_{ip}}} )^T}$ is the expression levels of $p$ target genes in sample $i$, and ${y_i}$ is the expression level of the TF gene in sample $i$. ${\beta _0}$ is the intercept and $\beta = {( {{\beta _1}, \cdots ,{\beta _p}} )^T}$ are the regulated regression coefficients. The TF gene regulates target gene $j$ if ${\beta _j} \ne 0\;( {j = 1, \cdots ,p} )$; the target gene $j$ and target gene $k$ are co-regulated by TF if both ${\beta _j} \ne 0$ and ${\beta _k} \ne 0$. ${\varepsilon _i}$ is independent and identically distributed random errors with mean 0 and variance ${\sigma ^2}$.

### Statistical selection methods

Based on the above statistical model, we developed four statistical selection methods to infer the potential TGs for a given TF and two methods to infer pathway regulatory networks based on the penalized regression model. The general objective function of the penalized regression model was defined as


(2) \begin{equation*}f\left( {\beta ;\lambda ,\alpha } \right) = L\left( {\beta ;y,x} \right) + P\left( {\beta ;\lambda ,\alpha } \right),\end{equation*}

where $L( {\beta ;{y_i},{x_i}} )$ is the loss function according to the observed expression levels of TGs and TF and $P( {\beta ;\lambda ,\alpha } )$ is the penalty function which can control the sparsity of the selected TGs.

#### Loss functions

In the above general objective function of the penalized regression model, we considered the following two loss functions, mean squared error (MSE) and Huber. The MSE loss function is defined as ${L^{MSE}}( {\beta ;y,x} ) = \frac{1}{{2n}}\mathop \sum \limits_{i = 1}^n {( {{y_i} - {\beta _0} - x_i^T\beta } )^2}$, which is very sensitive to outliers. The Huber loss function is defined as ${L^{Huber}}( {\beta ;y,x} ) = \mathop \sum \limits_{i = 1}^n {H_M}( {{y_i} - {\beta _0} - x_i^T\beta } )$, where ${H_M}( z )$ is the Huber function for an input value $z$, which is quadratic function for small $z$ values but grows linearly for large values of $z$. In this study, the parameter $M$ is defaulted to be one-tenth of the interquartile range (IRQ), as suggested by Deng et al.(21). For any given positive real $M$ (called shape parameter), the Huber function is defined as


(3) \begin{equation*}{H_M}\left( z \right) = \left\{ {\begin{array}{@{}*{2}{c}@{}} {{z^2}}&{\left| z \right| \le M}\\ {2M\left| z \right| - {M^2}}&{\left| z \right| >M} \end{array}} \right..\end{equation*}

#### Penalty functions

All of the three penalty functions we considered, Lasso, ENET and Net, contained the ${l_1}$ norm of the estimated effect sizes (${\beta _1}$). The ENET penalty is the combination of the ${l_1}{\mathrm{\;}}$norm and squared ${l_2}$ norm, ${P^{ENET}}( {\beta ;\lambda ,\alpha } ) = \lambda \alpha {\beta _1} + \frac{1}{2}\lambda ( {1 - \alpha } )\beta _2^2$. $\lambda >0$ and $\alpha \in [ {0,1} ]$ are the tuning parameters, where $\lambda$ controls the sparsity and $\alpha$ is the mixing proportion between *l*_1_ norm and *l*_2_ norm. The Lasso penalty is the special case of ENET ($\alpha = 1$) and ${P^{Lasso}}( {\beta ;\lambda ,\alpha } ) = \lambda {\beta _1}$, which only contains one tuning parameter $\lambda >0$. For the Net penalty, we assume that the genes involved in the same pathway are often co-regulated by a TF or the same regulatory mechanism, which is supported by previous studies (39–41). The Net penalty function can utilize prior biological network knowledge such as genetic pathways (26), which is a combination of the *l*_1_ norm and squared *l*_2_ penalty using the genetic network structure. As introduced in Kim and Sun (26), the ${P^{Net}}( {\beta ;\lambda ,\alpha } )$ is defined as


(4) \begin{eqnarray*} && {P^{Net}}\left( {\beta ;\lambda ,\alpha } \right) = \lambda \alpha {\beta _1} + \frac{1}{2}\lambda \left( {1 - \alpha } \right){\beta ^T}{{\bf{S}}^T}{\bf{LS}}\beta\nonumber\\ && \quad = \lambda \alpha \mathop \sum \limits_{j = 1}^p \left| {{\beta _j}} \right| + \frac{1}{2}\lambda \left( {1 - \alpha } \right)\mathop \sum \limits_{j = 1}^p \mathop \sum \limits_{j\sim k} {\left( {\frac{{{s_j}{\beta _j}}}{{\sqrt {{d_j}} }} - \frac{{{s_k}{\beta _k}}}{{\sqrt {{d_k}} }}} \right)^2}.\end{eqnarray*}

In formula (4), ${\bf{S}} = diag( {{s_1}, \cdots ,{s_p}} )$ is a diagonal matrix whose diagonal entries are the signs of estimated regression coefficients, which can be obtained from either the ordinary regression when $p \geq n$, or the ridge regression when $p \ge n$. It has been shown that the matrix ${\bf{S}}$ can accommodate the problem of failure of local smoothness between linked genes (42). For example, if two nearby target genes are negatively regulated by TF, the signs in their regression coefficients are expected to be different. In formula (4), ${\bf{L}}$ is a symmetric normalized Laplacian matrix, where the elements of ${\bf{L}}$,${L_{jk}}$, are given by


\begin{equation*}{L_{jk}} = \left\{ {\begin{array}{@{}*{2}{c}@{}} 1&{if\;j = k\;and\;{d_j} \ne 0}\\ { - {{\left( {{d_j}{d_k}} \right)}^{ - \frac{1}{2}}}}&{if\;j \ne k\;and\;j\sim k}\\ 0&{otherwise} \end{array}} \right.,\end{equation*}

where $j\sim k$ means that the target genes $j$ and $k$ are linked in the genetic network and ${d_j}$ is the total number of genes linked with the target gene $j$. Note that the genetic network information ${\bf{L}}$ are considered as the functional relationships among the target genes, which can be obtained from the existing annotation. For example, we can construct an association network using the pathways or biological processes information, where the targets genes are associated with each other if they are within a metabolic pathway or a biological process.

Based on the above two loss functions along with three penalty functions, we developed six statistical selection methods, MSE-Lasso, MSE-ENET, MSE-Net, Huber-Lasso, Huber-ENET and Huber-Net. For a given pair of $\lambda$ and $\alpha$, we can estimate the regression coefficients of $p$ target genes, $\beta$, by minimizing the objective function $f( {\beta ;\lambda ,\alpha } )$ introduced in formula (2). In other words, $\beta = {\mathrm{argmi}}{{\mathrm{n}}_\beta }f( {\beta ;\lambda ,\alpha } )$. The penalty function $P( {\beta ;\lambda ,\alpha } )$ is convex (26,43), so the solution to $\beta$ can be obtained via one of the convex optimization algorithms.

### Algorithm to solve the penalized regression models

Since $| {{\beta _j}} |$ is convex but not differentiable at ${\beta _j} = 0$ for $j = 1, \cdots ,p$, it is difficult to use the gradient descent method to find $\beta = {\mathrm{argmi}}{{\mathrm{n}}_\beta }f( \beta )$. Although we can use the general convex optimization solver CVX (44), it is too slow for real biological applications especially when there are a large number of genes involved in the analysis. Therefore, we adapted an accelerated proximal gradient descent (APGD) algorithm which is an effective algorithm when the objective function can be decomposed as a sum of a convex differentiable function and a convex non-differentiable function. In the six methods we developed, the objective function $f( \beta )$ can be decomposed as $f( \beta ) = g( \beta ) + h( \beta )$, where $g( \beta )$ is a convex differentiable function and $h( \beta )$ is a convex non-differentiable function. The idea behind APGD method is to make a quadratic approximation to $g( \beta )$ and leave $h( \beta )$ unchanged (45), then use the iterations to solve $\beta = {\mathrm{argmi}}{{\mathrm{n}}_\beta }f( \beta )$ (Details in the Supplemental Texts S3–S8).

### Selection probability

To obtain a stable selection result, we applied the stability selection method, namely, half-sample approach, to each target gene, which does not need to choose the optimal tuning parameters in selection methods. For a pair of fixed values of $\lambda$ and $\alpha$ ($\alpha = 1$ for Lasso penalty), $n/2$ samples are selected at random without replacement and then the regression coefficients are estimated based on this subset of samples. This process is repeated $B$ times for each pair of $\alpha$ and $\lambda$ over a grid set of $\alpha$ and $\lambda$. Let ${\hat \beta _j}( {{S_b};\alpha ,\lambda } )$ denote the estimated regression efficient for the $b$th sample (${S_b},\;b = 1, \cdots ,B$), the selection probability of target gene $j$, $S{P_j}$, is the maximum portion of non-zero ${\hat \beta _j}( {{S_b};\alpha ,\lambda } )$ over all pairs of $\alpha$ and $\lambda$. In other words,


(5) \begin{equation*}S{P_j} = \mathop {\max }\limits_{\alpha ,\lambda } \frac{1}{B}\mathop \sum \limits_{b = 1}^B I\left( {{{\hat \beta }_j}\left( {{S_b};\alpha ,\lambda } \right) \ne 0} \right),\end{equation*}

where $I( {{{\hat \beta }_j}( {{S_b};\alpha ,\lambda } ) \ne 0} )$ is an indicator function and $I( {{{\hat \beta }_j}( {{S_b};\alpha ,\lambda } ) \ne 0} ) = 1$ if ${\hat \beta _j}( {{S_b};\alpha ,\lambda } ) \ne 0$ for $b = 1, \cdots ,B$.

There are two major advantages for the use of selection probability. First, we avoid selecting the optimal tuning parameters $\lambda$ and $\alpha$, which is challenging in penalized regression analysis. Second, it has been shown that the results obtained from the half-sample approach and the selection probabilities are more stable than those obtained from the cross-validation (26,46). The main challenge of the stability selection method is how to appropriately choose the grid sets of the two parameters $ \lambda$ and $\alpha$. For a given $\alpha$, the smallest $\lambda$ such that all estimated coefficients are zeros from two loss functions, MSE and Huber, can be defined as


(6) \begin{equation*}\lambda _{max}^{MSE} = \mathop {\max }\limits_{j = 1, \cdots ,p} \left| {\mathop \sum \limits_{i = 1}^n \left( {{y_i} - {\beta _0} - {x_{ij}}{\beta _j}} \right){x_{ij}}} \right|/\alpha ,\end{equation*}

(7) \begin{equation*}\lambda _{max}^{Huber} = \mathop {\max }\limits_{j = 1, \cdots ,p} \left| {\mathop \sum \limits_{i = 1}^n \nabla {H_M}\left( {{y_i}} \right){x_{ij}}} \right|/\alpha ,\end{equation*}

where $\nabla {H_M}( {{y_i}} ) = 2{y_i}I( {| {{y_i}} | \le M} ) + 2Msign( {{y_i}} )I( {| {{y_i}} | >M} )$ is the gradient of Huber function. Therefore, the grid set of $\lambda$ can be set as a log_10_-scale from $ratio{\mathrm{*}}{\lambda _{max}}$ to ${\lambda _{max}}$, where the $ratio = 0.01$ as suggested by R package glmnet.

### Implementation

Six statistical selection methods based on the penalized regression models and the APGD algorithm for solving these six statistical methods had been implemented in both Python3 and R and then packed into TGPred. Both of them used commonly used libraries for scientific computing. For Python3 version of TGPred, we used numpy, scipy and sklearn to support efficient mathematical and dataframe computing, cvxpy to compare the runtime and estimated results of APGD with commonly used CVX, and networkx to generate synthetic data based on the BA network setting. For R version of TGPred, we used Matrix and MASS to support the efficient mathematical computing, and mvtnorm and igraph to generate synthetic data. TGPred can be directly used within Python and R. Both regulation effect ${\beta _j}$ and selection probability $S{P_j}$ of target gene $j$ can be calculated by TGPred for $j = 1, \cdots ,p$. Note that the large-scale genetic data set is acceptable to APGD and the computation time was evaluated on the high-performance computing (HPC) cluster (Intel Xeon E5-2670 2.6 GHz, 16 GB RAM). For example, when the number of target genes is >30 000 ($p >30\,000)$ and the half-sample approach with $B = 500$ times of resamplings was used, the computation times of ENET-based methods were about 12h CPU time with 90 pairs of tuning parameters $\alpha$ and $\lambda$; the computation times of Lasso-based methods were about 8h CPU time with 50 tuning parameters $\lambda$; and the computation times of Net-based methods were about 26 h CPU time with 90 pairs of tuning parameters $\alpha$ and $\lambda$. TGPred package has been made publicly available on GitHub as open-source software for downloading (https://github.com/xueweic/TGPred); more detailed information on how to install and run the tool was enclosed in the packages.

## RESULTS

### Validation of the methods with simulated data

Simulation studies were used to evaluate the performance of the six statistical selection methods we developed based on the penalized regression models. We considered three simulation settings, the general setting and two network settings, and we used $n = 300$ samples and $p = 500$ TGs in all simulation settings. For each simulation setting, the regulation effects for all genes based on each method were estimated by the improved APGD, and the selection probabilities were calculated by the half-sample approach with $B = 500$ times. Then, the true positive rates (TPRs) were used to evaluate the selection performance, which is defined as the number of the truly regulated genes among the selected top-ranked genes divided by the total number of truly regulated genes.

**Figure 1.**
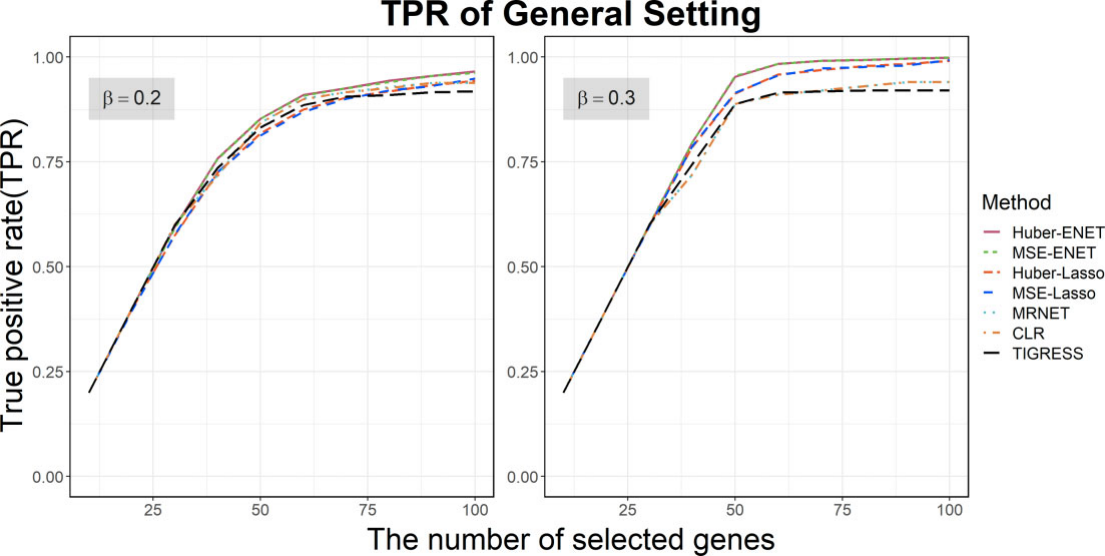
The true positive rates (TPRs) of the four statistical selection methods for identifying transcription factor (TF)–target gene (TG) relationships in the general setting. The selection probabilities were calculated using the half-sample approach method with $B = 500$ times of resampling. Each curve represents the mean of 100 simulations. The standard errors for all methods were too small, and were thus not plotted.

In the general setting, TGs were independent with each other. Therefore, we only compared the performances of Huber-Lasso, MSE-Lasso, Huber-ENET and MSE-ENET with the three comparison methods, CLR, MRNET and TIGRESS. Figure 1 showed the TPRs of these for methods based on the number of selected top-ranked genes. As it is known, the bigger pre-set regulation effect of $\beta$ may result in the higher TPRs of all methods, while the lower pre-set regulation effect may result in the lower TPRs. In both cases, we cannot differentiate the performances of different methods. Therefore, we pre-set the regulation effect $\beta = 0.2$ or $0.3$, and 50 TGs were regulated by a given TF in this simulation setting. When $\beta = 0.3$, all four methods performed equivalently well as we cut 40 top-ranked genes or less, achieved over 80% TPRs as we cut 50 top-ranked genes, and 95% TPRs as we cut 85 top-ranked genes. MSE-ENET and Huber-ENET performed better than the other five methods.

**Figure 2.**
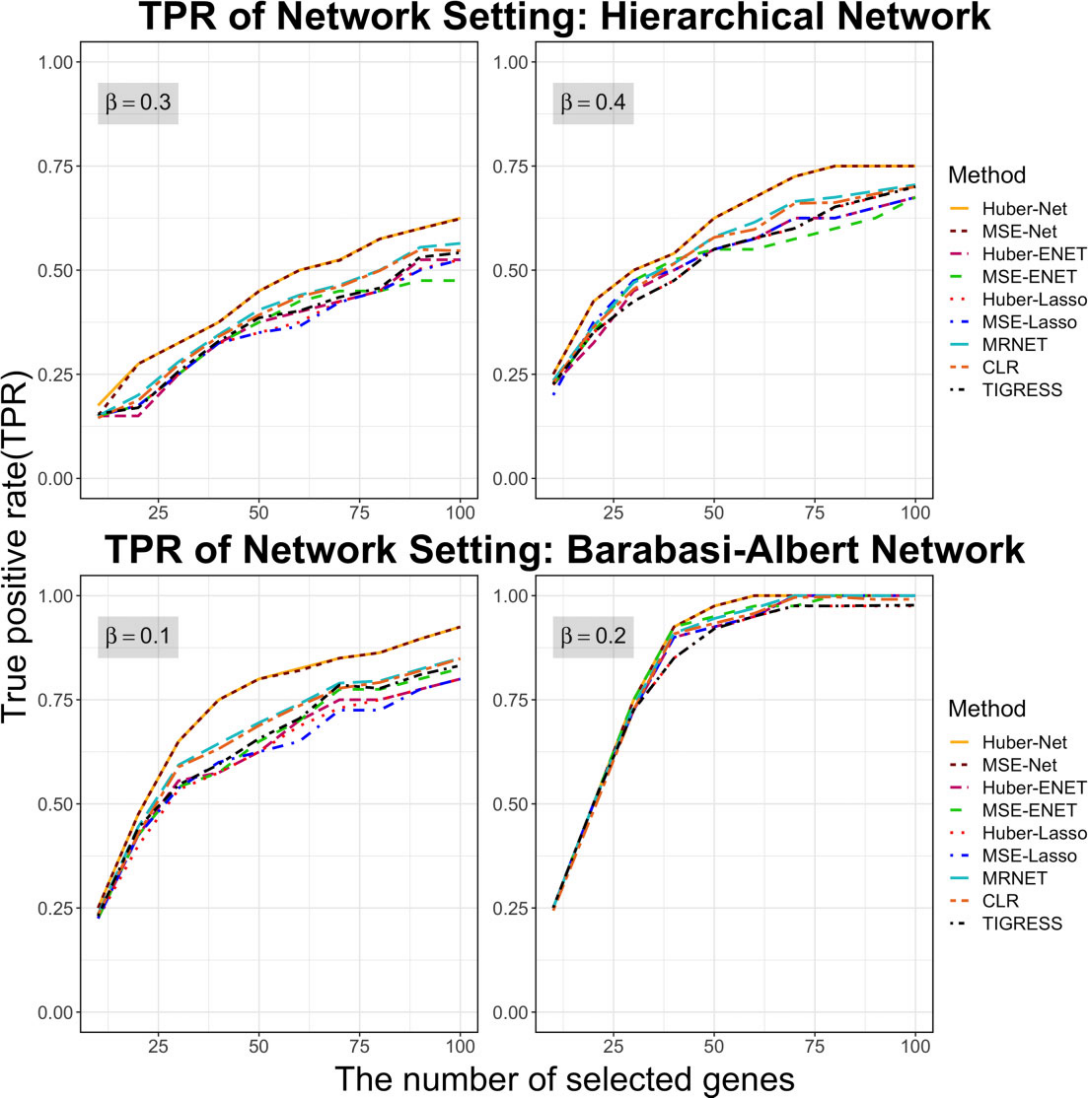
The true positive rates (TPRs) of the six statistical selection methods in the two network settings, the hierarchical network and the Barabasi-Albert network. The selection probabilities were calculated using the half-sample approach method with $B = 500$ times of resampling. Each curve represents the mean of 50 simulations. The standard errors for all methods were too small, and were not plotted.

For the network settings, we considered two network structures, the hierarchical network (Supplementary Figure S1) and the Barabasi-Albert network (not shown). Figure 2 showed how TPRs varied with the different numbers of the top-ranked genes for different methods. For the hierarchical network where 45 TGs (out of 500 genes) were truly regulated by a given TF, we pre-set the regulation effects $\beta = 0.3$ or $0.4.$ Since the Net penalty function incorporated the network structure, TPRs of Huber-Net and MSE-Net were higher than the other seven methods. For the Barabasi-Albert network setting where 40 true TGs (out of 500 genes) were regulated by a given TF, we pre-set the regulation effect $\beta = 0.1\;$or $0.2$. Huber-Net and MSE-Net also had the highest TPRs as expected, indicating that Huber-Net and MSE-Net can incorporate the functionally associated genes to increase the probability of these genes to be selected as the TGs for a given TF. Based on both TPRs, we concluded that Huber-Net and MSE-Net performed slightly better than MRNET and CLR and better than all other methods. Compared to the general setting, it is obvious that the four TF–TG identification methods performed less differentially in the two network settings, as shown in Figure 2.

We also compared the computational efficiency and the regression coefficients estimated by APGD and CVX, a commonly used package for convex optimization, for several pairs of tuning parameters $\lambda$ and $\alpha$. Figures S2–S4 showed that the computation times of CVX and APGD among all grid sets of $\alpha$ and $\lambda$ based on $B = 500$ subsamples drawn with the half-sample approach. Supplementary Figure S2 showed the computation times of Huber-Lasso, Huber-ENET, MSE-Lasso and MSE-ENET under the general setting with $\beta = 0.2$. For ENET penalty function, ${n_\lambda } = 1, \cdots ,10$ indicated the order of selected $\lambda$ in a log_10_-scale from $ratio*{\lambda _{max}}$ to ${\lambda _{max}}$, where ${\lambda _{max}}$ is related to $\alpha = 0.1, \cdots ,0.9$. For Lasso penalty, ${n_\lambda } = 1, \cdots ,100$ indicated the order of selected $\lambda$ in a log_10_-scale from $ratio*{\lambda _{max}}$ to ${\lambda _{max}}$, where ${\lambda _{max}}$ is related to $\alpha = 1$. The data sets were simulated under the same setting (Supplemental Text S1). All analyses were performed on a macOS (2.7 GHz Quad-Core Intel Core i7, 16 GB memory). APGD had much lower computational complexity than CVX since the running time of APGD was usually less than one fifth of that of CVX for four TF–TG identification methods (Supplementary Figure S2). A disadvantage of CVX is that all of the estimated regression coefficients were not equal to 0 (around ${10^{ - 22}}$ for non-zero regression coefficients). Therefore, the stability selection method may not be applicable to the CVX method since it is difficult to find a cut-off threshold for the regression coefficients. The APGD algorithm was also evaluated under the hierarchical network and Barabasi-Albert network settings for all six methods. As shown in Figures S3-S4, the computation times of APGD were much shorter than those of CVX no matter which methods (Huber-Lasso, Huber-ENET, Huber-Net, MSE-Lasso, MSE-ENET and MSE-Net) it was applied to. The results manifested the consistent lower computational complexity of APGD than CVX, as we had observed for the general setting (Supplementary Figure S2).

We compared the regression coefficients estimated by APGD and CVX for several pairs of tuning parameters $\lambda$ and $\alpha$. Figures S5–S7 showed that the QQ plots of the regression coefficients estimated by both CVX and APGD. Supplementary Figure S5 showed the estimation of regulation effects of Huber-Lasso, Huber-ENET, MSE-Lasso and MSE-ENET under the general setting with $\beta = 0.2$. The values lied along the diagonal line as the Huber loss function was used, indicating the regression coefficients estimated by CVX and APGD were identical. When the MSE loss function was used, the non-zero estimations of regulation effects of CVX were greater than those of APGD (Supplementary Figure S7). However, there were only 50 true TGs (out of 500 genes) that were regulated by a given TF in this simulation setting. That is, CVX obtained more false positives than APGD. Except for those false positives estimated by CVX, the regression coefficients estimated by these two methods were nearly identical. Figures S6-S7 showed that the estimation of regulation effects of our proposed six statistical selection methods under the network setting, where we used $\beta = 0.4$ in the hierarchical network setting (Supplementary Figure S6) and $\beta = 0.1$ in the Barabasi-Albert network setting (Supplementary Figure S7). We observed that the patterns of the estimation performance were similar to those shown in Supplementary Figure S5.

### Validation of the four TF–TG identification methods with SND1 overexpression transcriptomic data

**Figure 3.**
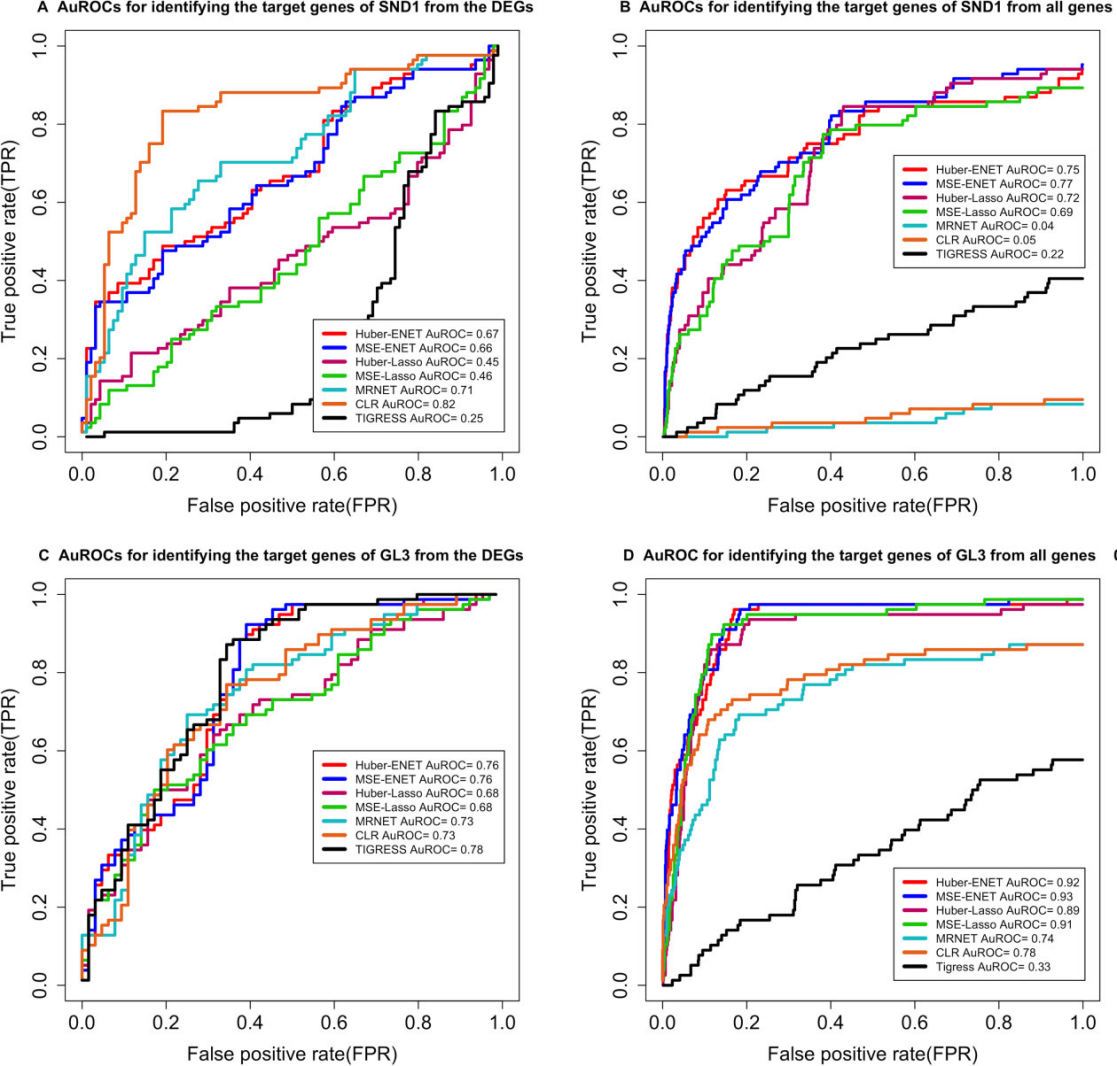
The performance of four transcription factor (TF)–target gene (TG) identification methods. (**A**) ROCs generated with the SND1 overexpression transcriptomic data set of 178 differentially expressed genes (DEGs) (resulting from SND1 overexpression) from *Populus trichocarpa*. (**B**) ROCs generated with the SND1 overexpression transcriptomic data set of all genes (33 691) from *Populus trichocarpa*. (**C**) ROCs generated with the gl3 overexpression transcriptomic data set of 144 DEGs (resulting from *gl3* overexpression) from *Zea mays*. (**D**) ROCs generated with the *gl3* overexpression transcriptomic data set of all genes (30 263) from *Zea mays*. AuROC, area under the receiver-operating characteristic curve.

178 DEGs in response to *SND1* overexpression were identified in our early publication (16). These 178 DEGs were classified into two groups, 84 direct target genes and 94 indirect target genes of SND1 using Top-down GGM Algorithm. Of these 84 direct target genes, 16 randomly drawn genes were tested with ChIP-PCR assay using SND1 antibodies, all of them were proven to be the true direct target genes of SND1(16). Sixteen genes randomly drawn from these 94 indirect target genes were also subjected to ChIP-PCR using SND1 antibodies and 15 were proven to be indirect target genes of SND1, indicating the 84 genes can be assumed to be true target genes of SND1 for testing our methods. Using the three time-point *SND1* overexpression transcriptomic data sets, we tested our methods for identifying these 84 direct target genes of SND1 from 178 DEGs and all 33691 genomic genes based on the selective probabilities yielded from each method. The results, as shown in shown in Figures 3A and B, demonstrated the following: (i) When applied to 178 DEGs, Huber-ENET and MSE-ENET in overall identified less positive TGs than CLR and MRNET methods but more than Huber-Lasso, MSE-Lasso and TIGRESS; Huber-Lasso, MSE-Lasso identified more positive genes than TIGRESS. Based on ROCs, Huber-ENET and MSE-ENET appeared to rank more positive genes to the very top of TG list than any other methods. (ii) When applied to all 33 691 genomic genes, Huber-ENET and MSE-ENET identified more positive genes than Huber-Lasso, and MSE-Lasso, while Huber-Lasso and MSE-Lasso identified more positive TGs than CLR, MRNET and TIGRESS.

### Validation of the four TF–TG identification methods with *glossy (gl3)* overexpression transcriptomic data

We also validated our methods with ***gl3* overexpression transcriptomic data** that was generated with Transcriptional-Activator Like effectors (TALes) system. Two TALes, dT1 and dT2, were constructed to target two non-overlapping 16-bp regions (4 and 48 bp upstream of the transcription start site) in the *gl3*’s promoter to activate it. Analysis of RNA-seq data yielded at 24 h revealed 144 DEGs (93 upregulated and 51 downregulated genes), that were activated by both dT1 and dT2 (25). From these 144 genes, we identified 93 tightly responsive genes to *gl3* and 78 direct TGs of *gl3* using top-down GGM Algorithm with a cut-off threshold of corrected *P*-values <0.05. The 78 genes contain 6 of 9 known glossy genes present in the literature, supporting that the 78 genes are true positive TGs. Using time-point *gl3* overexpression transcriptomic data sets, we tested our methods for identifying these 78 direct TGs of gl3 from 144 DEGs and all 30 263 genomic genes based on the selective probabilities. The results, as shown in Figures 3C and D, demonstrated the following: (i) when applied to 144 DEGs, Huber-ENET and MSE-ENET in overall identified less positive genes than TIGRESS but more than CLR and MRNET. Huber-Lasso, MSE-Lasso, underperformed all other methods; (ii) when applied to all 30 263 genomic genes, Huber-ENET and MSE-ENET outperformed all other methods because they identified more positive genes than Huber-Lasso and MSE-Lasso, while Huber-Lasso and MSE-Lasso identified more positive TGs than CLR, MRNET and TIGRESS.

### Validation of the two net-based methods for identifying lignin pathway genes and GRN

Maize B73 compendium transcriptomic data of 736 samples was used for predicting the regulatory relationships between transcription factor (TFs) and pathway genes (PWGs). A total of 2539 PWGs belonging to 446 pathways were obtained after the PWGs with 90% or more expression values being zero were removed. The Laplacian matrix ${\bf{L}}$ of 2539 PWGs was constructed based on 446 pathways, that is, two PWGs were associated together if they belong to at least one of 446 pathways. Since these 23 TFs are the known TFs that regulate lignin pathway in multiple plant species (34–38). We specifically examined 21 pathway genes in maize which were curated by Plant Metabolic Pathway (47) as lignin pathway genes.

**Figure 4.**
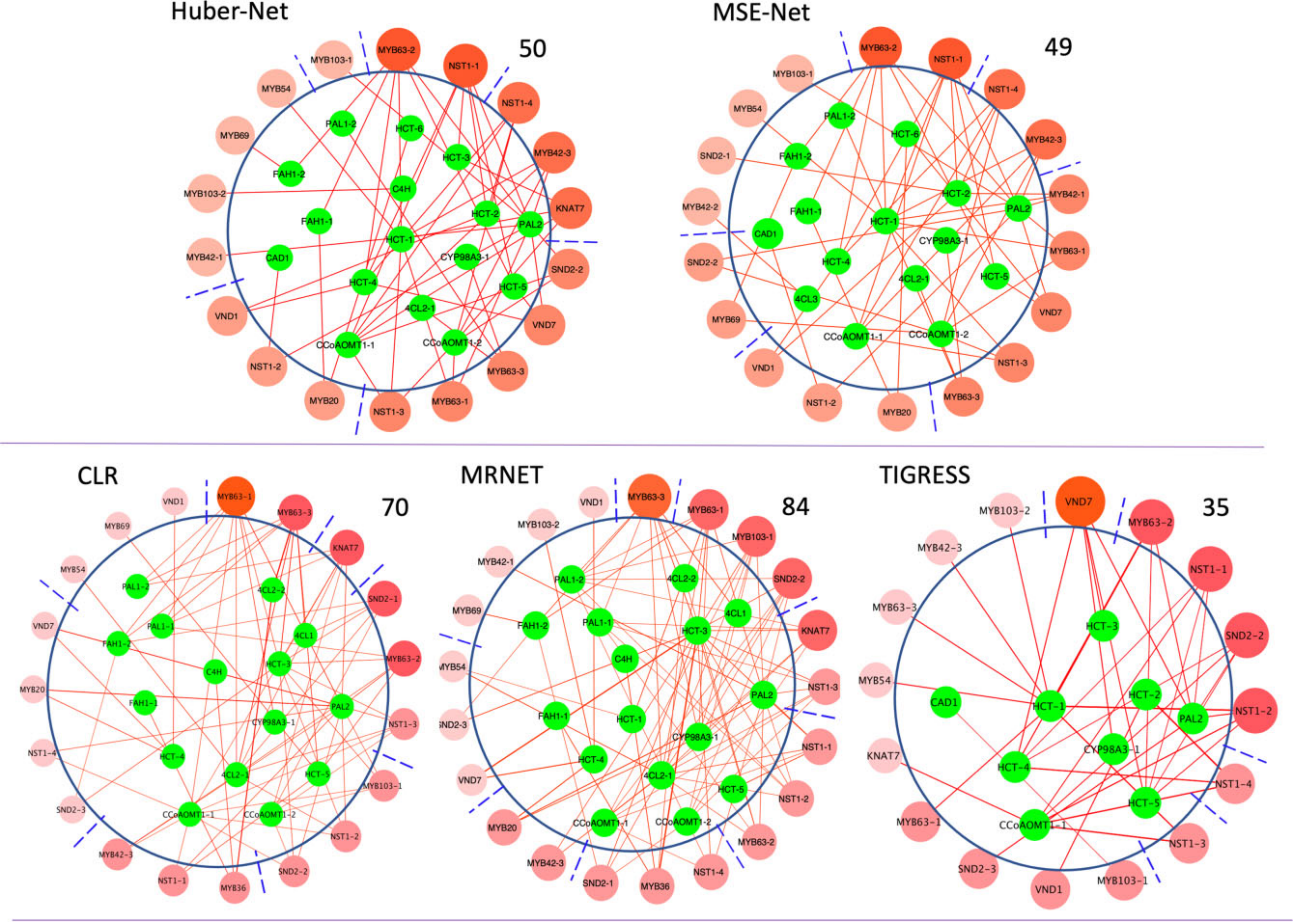
The gene regulatory networks of lignin pathway produced by Huber-Net and MSE-Net methods, with three network construction methods, CLR, MRNET and TIGRSS, used as comparison. The transcription factors (TFs) were ranked based on their connectivity to lignin pathway genes in clockwise. The number shown near each network is the number of edges identified by each method.

We applied Huber-Net and MSE-Net to two input subsets of transcriptomic data: one contains $2539$ PWGs × 736 samples, and the other contains 23 TFs × 736 samples to calculate the selection probability of 2539 PWGs to each of 23 TFs. For Huber-Net, nine $\alpha {\mathrm{\;}}$values ($\alpha = 0.1,0.2, \cdots ,0.9$) and 10 different $\lambda$ values in a calculated range from the loss function (‘Lambda_gird’ function from our developed package ‘APGD’) were used. For Huber-Lasso regression model, 100 $\lambda$ values in a calculated range from the loss function with $\alpha = 1$ were used. Furthermore, the parameter $B$, which represents the number of subsets of samples randomly drawn during the half-sample resampling, was set to 500. The resulting selection probabilities of the 2539 PWGs $ \times$ the 23 TFs calculated by Huber-Net and MSE-Net were shown in Tables S2 and S3, respectively, and the results by the three comparison methods, CLR, MRNET and TIGRESS, were shown in Tables S4, S5 and S6, respectively. We then extracted the selection probabilities of the 21 lignin PWGs $ \times$ the 23 TFs resulting from all methods, and were shown in Table S7. Since the comparison methods, CLR, MRNET and TIGRESS, use different statistics to evaluate the regulatory strengths, we could not use the same criterion to cut off the ranked PWGs to each TF. We hypothesized that the top 100 genes identified from 2539 PWGs for each TF by each method are its putative TGs, and summarized the TGs for all 23 TFs for each method. We then extracted the TF–TG pairs where the TGs are lignin pathway genes and compared which method could identify more regulatory relationships between the lignin pathway genes and the 23 TFs. The results are shown in Figure 4, where TFs were ranked clockwise based on the number of their connectivity to pathway genes; the TFs with higher connectivity are assumed to regulate more pathway genes and/or have larger impact on pathway genes and thus were ranked highly. The results showed that Huber-Net and MSE-Net methods identified 50 and 49 regulatory relationships, respectively, more than that of TIGRESS. The high number of regulatory relationships is the suggestive of a potential recognition of known regulatory relationships. However, both our methods identified less regulatory relationships compared to MRNET and CLR, which identified 84 and 70 regulatory relationships, respectively. Currently, we do not know which method is better than another. This is because we know these 23 TFs are lignin pathway regulators but exactly which pathway genes are the true targets of which TF are mostly unknown, and we thus cannot draw further and more firm conclusions that our methods are better or worse than comparison methods. Nevertheless, if the objective is to identify pathway regulators, the top TFs ranked for lignin pathway by different methods shared many TFs in common. We also cut off the ranked PWG list to each TF generated by two Net-based methods using a selection probability threshold of 0.9, and the results are shown in Supplementary Figure S8, Huber-Net and MSE-Net identified 76 and 28 regulatory relationships, respectively. The ranking order of TFs changed slightly as compared to same methods as shown in Figure 4.

**Figure 5.**
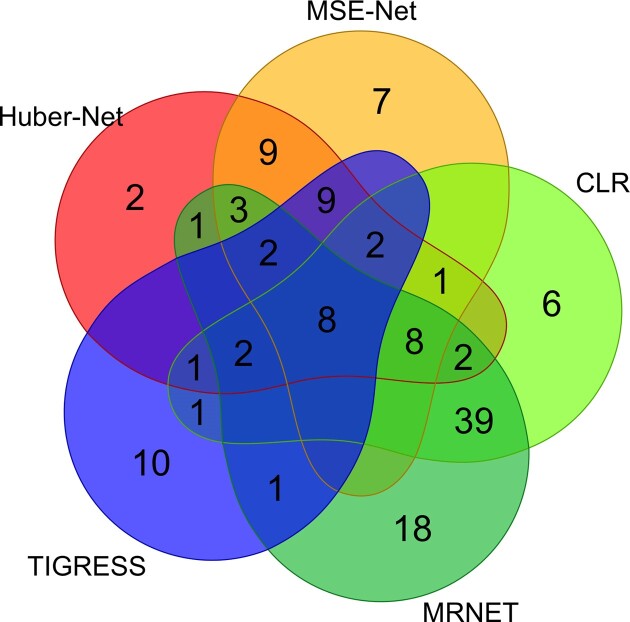
The Venn diagram that shows the overlaps of regulatory relationships between 23 transcription factors and lignin pathway genes identified by Huber-Net and MSE-Net, CLR, MRNET and TIGRESS methods.

Huber-Net identified the unique pathway genes that were not identified by any other methods including the three comparison methods. For example, C4H regulated by MYB103-2, and HCT-1 by KNAT7. MSE-Net uniquely identified CCoAOMT1-2 regulated by MYB69, 4CL3 by MYB42, HCT-1/2/3 by MYB42. Huber-Net and MSE-Net together uniquely identified FAH1-1, FAH1-2 by MYB63. To show the overlaps of the regulatory relationships identified by different methods, we generated a Venn diagram (Figure 5) based on the regulatory relationships shown in Figure 4. The results indicated that Huber-Net and MSE-Net are very similar methods because the gene regulatory relationships identified by the two methods had 42 in common. Similarly, MRNET and CLR are very similar methods because the gene regulatory relationships identified by two methods had 62 in common (Figure 5). In addition, of the 36 regulatory relationships identified by TIGRESS, 24 overlapped those identified by Huber-Net and/or MSE-Net, while only 17 overlapped those identified by MRNET and/or CLR, indicating it is more similar to Huber-Net and MSE-Net methods rather than MRNET and CLR (Figure 5). Since 24, 26 and 24 out of 50 regulatory relationships identified by Huber-Net overlapped those identified MRNET, CLR and TIGRESS, respectively, while 21, 19 and 21 out of 49 regulatory relationships identified by MSE-Net overlapped those identified MRNET, CLR and TIGRESS, respectively (Figure 5), Huber-Net and MSE-Net have their values in identifying unique true regulatory relationships.

## DISCUSSION

### Solving convex optimization problem by implementing APGD

The loss functions and the penalty functions we used in this study are all convex functions (26,39). Although CVX is the software commonly used for solving convex optimization problems(44), but one overt problem of CVX is its slowness when being used for large datasets. In this study, we implemented an APGD algorithm (45) to replace the CVX. APGD is an effective algorithm to solve an optimization problem with a decomposable objective function. Our simulation studies have shown that APGD was not only capable of obtaining the similar estimated regulation effects of all TGs for a given TF, but it also shortened the computation time to 1/5 of that by using CVX, enabling the prediction of true TGs of a TF from a large number of candidate TG genes (e.g. >30 000 as demonstrated in Supplementary Figure S2). In principle, CVX cannot be used to calculate the stable selection probability. Stable selection probability is calculated based on the ratio of the number of non-zero estimated regulation effects of a TG to the number of times we resampled in the half-sample approach, and all candidate tuning parameters. When using APGD, we can obtain a subset of TGs with non-zero regulation effects, and the rest subsets of TGs with zero regulation effects. Therefore, we do not need to choose threshold by applying APGD to the half-sample approach.

### Development and elucidation of six novel methods for identifying TGs of a TF

With the improved new APGD algorithm, we set out to develop novel methods to predict the TGs of a TF of interest using omics data, an important issue that has not been resolved in current bioinformatics. Using two loss functions, Huber and MSE, and three penalty functions, Lasso, ENET and Net, we developed four statistical selection methods, MSE-ENET, Huber-ENET, MSE-Lasso and Huber-Lasso for identifying TF–TG relationships, and two additional methods, MSE-Net and Huber-Net, for building pathway GRNs. The Huber loss function, which is a hybrid of squared errors for relatively small errors and absolute errors for relatively large errors (see Formula (3)), has been shown to be more robust than MSE loss function when there are outliers (48). To test the four TF–TG identification methods, we used the synthetic data generated from the general setting and found that ENET-based methods performed better than Lasso-based methods if all TGs are independent (Figure 1). When the two network settings were used to test all six methods and the three comparison methods, the MSE-Net and Huber-Net outperformed the other four non-Net methods since the Net penalty could incorporate the network structure of TGs for enhancing the prediction. Notably, one tuning parameter $\lambda$ from Lasso penalty and two tuning parameters $\alpha$ and $\lambda$ from ENET or Net penalty were obtained from the cross-validation by minimizing the predicted accuracy (21,49). However, the results are not stable since the samples were randomly split in the cross-validation (26). Fortunately, a stability selection method has been developed by Meinshausen and Bühlmann (46) that uses a subsampling approach to obtain a stable selection result; this subsampling approach can be used to determine the amount of regularization. In this study, we employed the selection probabilities to evaluate and select candidate TGs of a given TF.

**Figure 6.**
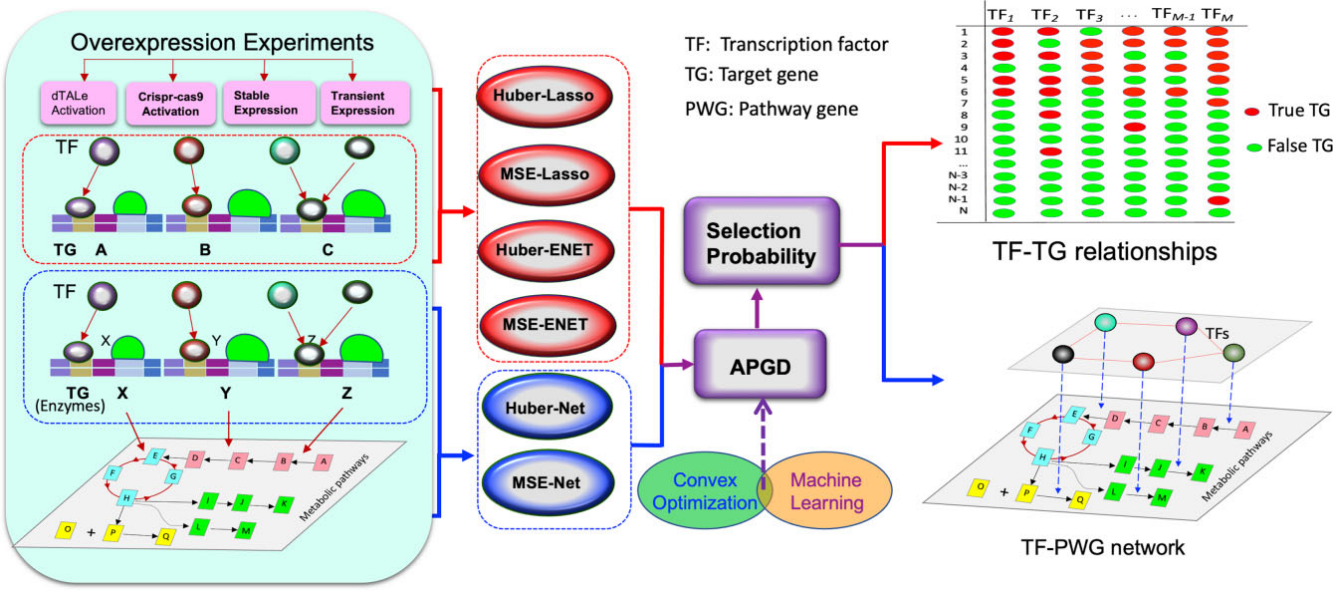
An integrative framework for identifying target genes (TGs) of a transcription factor (TF) of interest using transcriptomic data by integration of statistics, machine leaning and convex optimization.

Plant biologists frequently employ a transient system or develop transgenic lines to perturb a TF, as shown in Figure 6; (i) this is followed by RNA-seq experiments to obtain the transcriptomic data; after the DEGs pertaining a given TF are identified, biologists usually selected some DEGs based on their significant levels (e.g. corrected *P*-values or *q*-values) to validate their functions; (ii) using a correlation method (50), a dependence-based method (11,51) or a modeling method to identify candidate genes to validate (52); (iii) using top-down GGM algorithm (16,17,53,54) to predict TGs of the TF from the DEGs; However, correlation and independence-based methods usually have a low accuracy and top-down GGM algorithm suffers from constraint to scaling up due to the high computational cost of searching the space of a complete combination of a subset of candidate genes. For this reason, there is a pressing need to develop efficient methods for identifying the true TGs of a given TF. In addition, there are some other circumstances where we need new methods to identify or validate the TGs of a TF. For example, when genome-wide experiments like ChIP-seq and DAP-seq are conducted to identify putative TGs of a TF, a few hundred to even twenty thousand putative TGs can be identified, indicating that their promoters contain TF binding site. However, the presence of a TF-binding site of the TF does not necessarily guarantee an activation. We need highly efficient methods to validate the existence of an effect-and-response relationship between the TF and the putative TGs identified by ChIP-/DAP-seq. In this sense, our methods, Huber-ENET, Huber-Lasso, MSE-ENET and MSE-Lasso, fill in a gap of lacking an effective method for predicting and/or validating TGs of a TF of interest using large-scale omics data. Such methods are sought by many biologists. Our methods resampled a large number of subsets of data (e.g. 500) to compute the selection probabilities of all genes to one TF simultaneously, and then selected top-ranked TGs based on the stabilities of selection probabilities across all subsets. Therefore, our methods augmented the selection process and increased the reliability of TGs. Even if each time we computed linear relationships of one TF with all genomic genes or the DEGs resulting from the TF overexpression with one re-sampled subset, the aggregation of the selection probabilities from all sampled subsets could increase the chance of the nonlinear true relationships being captured.

Instead of identifying TGs of a TF independently, we sometimes need to investigate if a TF regulates a pathway or a biological process. In this case, we can determine if a TF’s TGs contain multiple genes belonging to a pathway or a gene ontology that represents a biological process. Toward this goal, we developed Huber-Net and MSE-Net methods based on a network-based penalty. In our extensive simulation studies based on the network setting, we showed that Huber-Net and MSE-Net performed better than the other four methods in terms of the true positive discovery rates. We then applied these two methods to identify true TGs of 23 TFs, which are known to regulate lignin pathway (34–38), from all 2539 pathway genes (PWGs) of maize.

### The power of statistics, machine learning and optimization combined approaches

In this study, we combined statistics (half-sample approach-derived selection probability), machine learning (regularization in unsupervised learning) and convex optimization (solving regularization with APGD) to identify TGs of a TF of interest, the flowchart depicting the methods is illustrated in Figure 6. Our results showed that this kind of combined approach has higher efficacy in identifying the true TGs, as we shown early (21).

In our methods, we utilized two loss functions. The Huber loss function is a combination of linear and quadratic loss functions, and the MSE loss function, which measures the average of the squared errors, ensures that our trained model has no outlier predictions with huge errors. MSE puts more weights on these errors due to the squared portion of the function. The mathematical benefit of MSE is particularly evident in its use at analyzing the performance of linear regression, as it allows one to partition the variation in a dataset into variation explained by the model and variation explained by randomness. Huber loss is more robust to outliers than MSE loss and least absolute deviation (LAD) loss, and has higher statistical efficiency than the LAD loss function in the absence of outliers (48).

In addition, we utilized three different penalty functions: Lasso, ENET and Net. Lasso penalty adds a penalty for non-zero coefficients to penalize the sum of their absolute values (${l_1}$ penalty). As a result, for high values of $\lambda$, many coefficients are exactly zero under Lasso. ENET penalty was proposed in response to the critique that the the variable selection of Lasso only considers the absolutely value of estimated effects, resulting in instability. ENET penalty combines the penalties of ridge regression and Lasso to gain ‘super-penalty’. Net penalty can incorporate a set of genes like a pathway or a biological process as represented by a gene ontology, and enables us to investigate if a TF regulates multiple genes involved in a pathway or a biology process. When TGs of multiple TFs are predicted, we can then use the results to screen the TFs for regulation of a specific metabolic pathway, biological process and complex trait.

We demonstrated that the improved APGD had less computational complexity for solving the convex optimization problem with both differentiable and undifferentiable functions. Traditional regularization methods need to choose optimal tuning parameters. One limitation of traditional regularization methods with cross-validation is that they depend on the saturation of the data; different data sets may obtain different tuning parameter sets, leading to different or unstable results. APGD is a highly efficient approach to solve our proposed methods as well as the other penalized regressions.The incorporation of half-sample-based selection probability allows for more stable results and avoids the choice of optimal tuning parameters. Therefore, integration of statistics, machine learning and optimization enables us to take the advantage of all methods and combines them to generate a powerful approach to identify true TGs of a TF with high efficacy.

Due to the disadvantage of the feature selection procedure, we cannot check if the selected genes have strong evidence related to the outcome. For future studies, we plan to integrate statistical inference in the selection procedure and further investigate the selection performance by integrating both selection and statistical inference.

## CONCLUSIONS

Four new statistical selection methods, referred to as Huber-ENET, MSE-ENET, Huber-Lasso and MSE-Lasso for identifying TGs of a TF of interest and two new methods, Huber-Net and MSE-Net, for inferring pathway GRNs have been developed by integration of statistics, machine leaning and convex optimization approaches. An APGD algorithm was developed to solve convex optimization with significantly reduced computation times. Comprehensive simulations and analyses of the four TF–TG identification methods using synthetic data under a general setting indicated that Huber-ENET, MSE-ENET, Huber-Lasso and MSE-Lasso could be used to identify true TGs of a TF with high efficacy, especially in genome-wide predictions. In simulations using the data from two network settings, Huber-Net and MSE-Net outperformed any other non-Net methods for identifying true TGs involved in a pathway or biological process. For real data, the Huber optimization has a noticeable contribution to the identification of true TGs of a given TF by increasing the selection probabilities as compared to MSE, and Huber-Net and MSE-Net could identify many unique regulatory relationships as compared to CLR, MRNET and TIGRESS. The ENET penalty function contributed greatly to enhancement of the method efficacy as compared to Lasso. AuROC plotting showed that all six methods could rank more positive known TGs to top of TG lists. The TF–TG identification methods developed will fill a methodological gap for genome-wide TF–TG prediction and have a great potential for being used to validate ChIP/DAP-seq results, while the Net-based methods will be instrumental for inferring pathway GRN.

## Supplementary Material

lqad083_Supplemental_Files

## Data Availability

The R package TGPred and Python module TGPred 52 are freely and publicly available at: https://zenodo.org/record/8297854 (R version) and https://zenodo.org/record/8299724 (Python version).
