# Supplementary material for: TGPred: efficient methods for predicting target genes of a transcription factor by integrating statistics, machine learning and optimization
Source: NAR Genom Bioinform. 2023 Sep 13;5(3):lqad083. doi: 10.1093/nargab/lqad083 (PMC10498345; doi:10.1093/nargab/lqad083)
Supplement: lqad083_Supplemental_Files [file lqad083_supplemental_files.zip › Suppl_Materials5.31.2023.pdf]

Supplemental Materials

TGPred: Efficient methods for predicting target genes of a transcription factor by integrating statistics, machine learning, and optimization

Xuewei Cao[1,§] , Ling Zhang[2,3,§], Md Khairul Islam[2, 3], Mingxia Zhao[4], Cheng He[4], Kui Zhang[1], Sanzhen Liu[4], Qiuying Sha[1,*] and Hairong Wei[1,2,3,*]

[1] Department of Mathematical Sciences, Michigan Technological University, Houghton, Michigan 49931, United States of America.

[2] Computational Science and Engineering Program, Michigan Technological University, Houghton, Michigan 49931, United States of America.

[3] College of Forest Resources and Environmental Science, Michigan Technological University, Houghton, Michigan 49931, United States of America.

[4] Department of Plant Pathology, Kansas State University, Manhattan, Kansas 66506, United States of America.

[#] Both authors contributed equally

[§] Corresponding authors:

Hairong Wei, College of Forest Resources and Environmental Science, Michigan Technological University, Houghton, Michigan 49931, USA. E-mail: hairong@mtu.edu

Qiuying Sha, Department of Mathematical Sciences, Michigan Technological University, Houghton, Michigan 49931, USA. E-mail: qsha@mtu.edu

Outline

Supplemental Figures

i. **Figure S1.** The hierarchical network module is used in the hierarchical network setting. There is a total of 100 genes that contain a centered gene.
ii. **Figure S2.** The computation times of CVX versus APGD in the general setting ($\beta = 0.2$) among all grid sets of α and λ based on $B = 100$ half-sample approach.
iii. **Figure S3.** The computation times of CVX versus APGD in the hierarchical network setting ($\beta = 0.4$) among all grid sets of α and λ based on $B = 100$ half-sample approach.
iv. **Figure S4.** The computation times of CVX versus APGD in the Barabasi-Albert network setting ($\beta = 0.1$) among all grid sets of α and λ based on $B = 100$ half-sample approach.
v. **Figure S5.** The estimation of regulation effects (beta) comparison of CVX versus APGD in the general setting ($\beta = 0.2$) by different algorithms.
vi. **Figure S6.** The estimation of regulation effects (beta) comparison of CVX versus APGD in the hierarchical network setting ($\beta = 0.4$) by different algorithms.
vii. **Figure S7.** The estimation of regulation effects (beta) comparison of CVX versus APGD in the Barabasi-Albert network setting ($\beta = 0.1$) by different algorithms.
Figure S8. The gene regulatory networks of lignin pathway genes produced by the two Net methods, Huber-Net and MSE-Net, where pathway gene list for each TF was cut off with selection probability equals or larger than 0.9. The transcription factors are ranked in descending order by their connectivity to pathway genes in clockwise. The input data contained two subsets of transcriptomic data from maize B73**:** one was 2539 PWGs x 736 samples, and the other was 23 TFs x 736 samples.

Supplemental Texts

i. **Text S1.** The general simulation settings.
ii. **Text S2.** Simulation settings if the target genes have the biological network structure.
iii. **Text S3.** APGD algorithm to solve Huber-Lasso.
iv. **Text S4.** APGD algorithm to solve Huber-ENET.
v. **Text S5.** APGD algorithm to solve Huber-Net.
vi. **Text S6.** APGD algorithm to solve MSE-Lasso.
vii. **Text S7.** APGD algorithm to solve MSE-ENET.
viii. **Text S8.** APGD algorithm to solve MSE-Net.

Supplemental Tables Legends

i. **Table S1.** Accession identifiers of maize B73 compendium transcriptomic data set with 736 RNA-seq samples.

ii. **Table S2.** Selection Probabilities of 23 TFs with 2539 PWGs by Huber-Net. A red highlight is a pathway gene, while a blue highlight indicates the pathway gene was among the top 100 genes to a TF ranked by the method
iii. **Table S3.** Selection Probabilities of 23 TFs with 2539 PWGs by MSE-Net. A red highlight is a pathway gene, while a blue highlight indicates the pathway gene was among the top 100 genes to a TF ranked by the method
iv. **Table S4.** Selection Probabilities of 23 TFs with 2539 PWGs by CLR. A red highlight is a pathway gene, while a green highlight indicates the pathway gene was among the top 100 genes to a TF ranked by the method
v. **Table S5.** Selection Probabilities of 23 TFs with 2539 PWGs by MRNET. A red highlight is a pathway gene, while a green highlight indicates the pathway gene was among the top 100 genes to a TF ranked by the method
vi. **Table S6.** Selection Probabilities of 23 TFs with 2539 PWGs by TIGRESS. A red highlight is a pathway gene, while a blue highlight indicates the pathway gene was among the top 100 genes to a TF ranked by the method
vii. **Table S7.** Selection probabilities of 23 TFs with 21 phenylpropanoid PWGs by six novel statistical selection methods.

References

Supplemental Figures

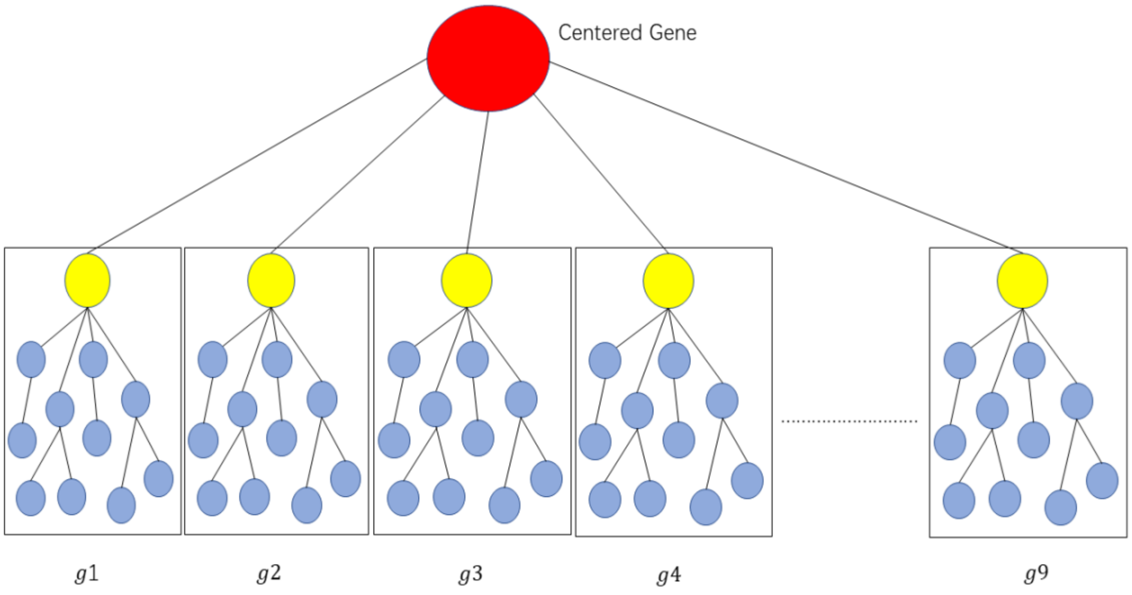

Figure S1. The hierarchical network module is used in the hierarchical network setting. There is a total of 100 genes that contain a centered gene.

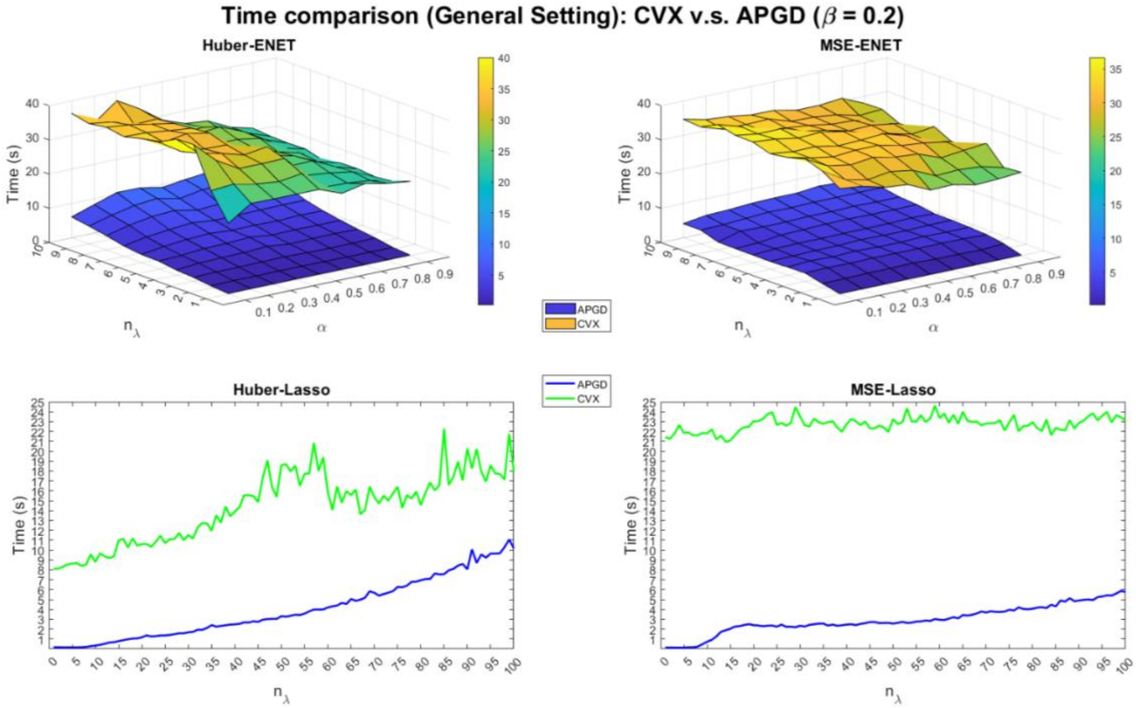

Figure S2. The computation times of CVX versus APGD in the general setting ($\beta = 0.2$) among all grid sets of α and λ based on half-sample approach with $B = 500$ times of resampling.

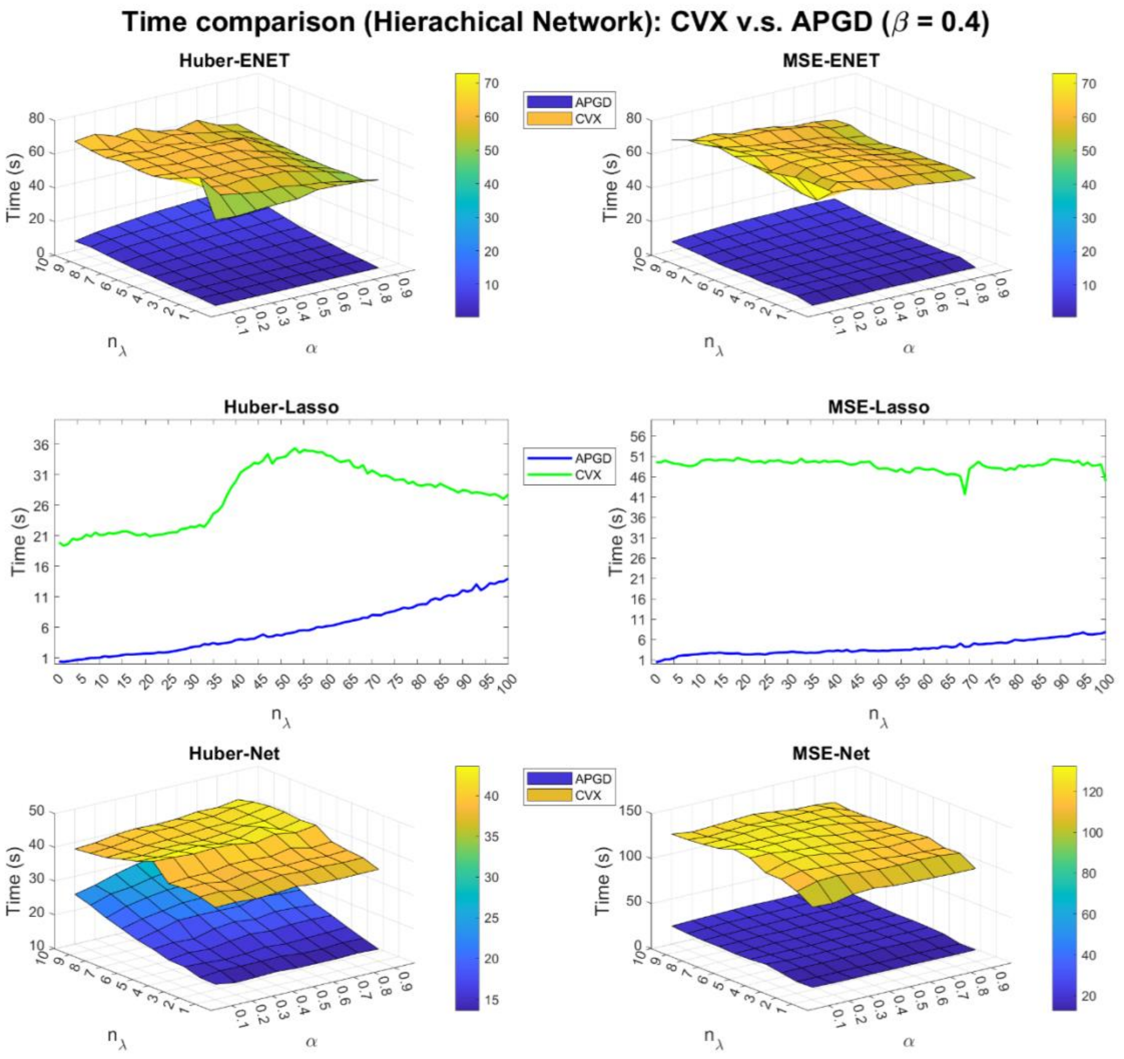

Figure S3. The computation times of CVX versus APGD in the hierarchical network setting ($\beta = 0.4$) among all grid sets of α and λ based on half-sample approach with $B = 500$ times of resampling.

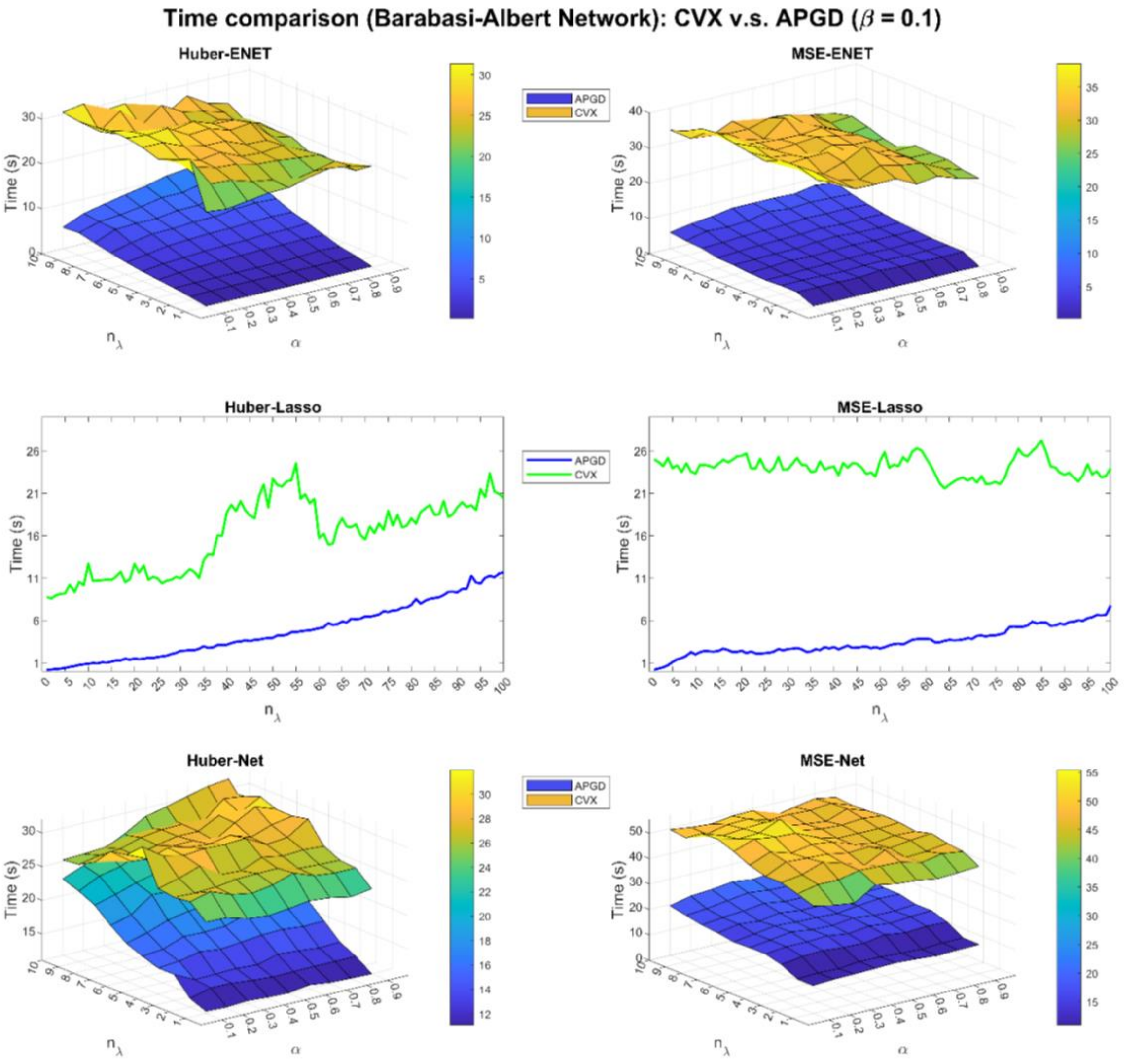

Figure S4. The computation times of CVX versus APGD in the Barabasi-Albert network setting ($\beta = 0.1$) among all grid sets of α and λ based on half-sample approach with $B = 500$ times of resampling.

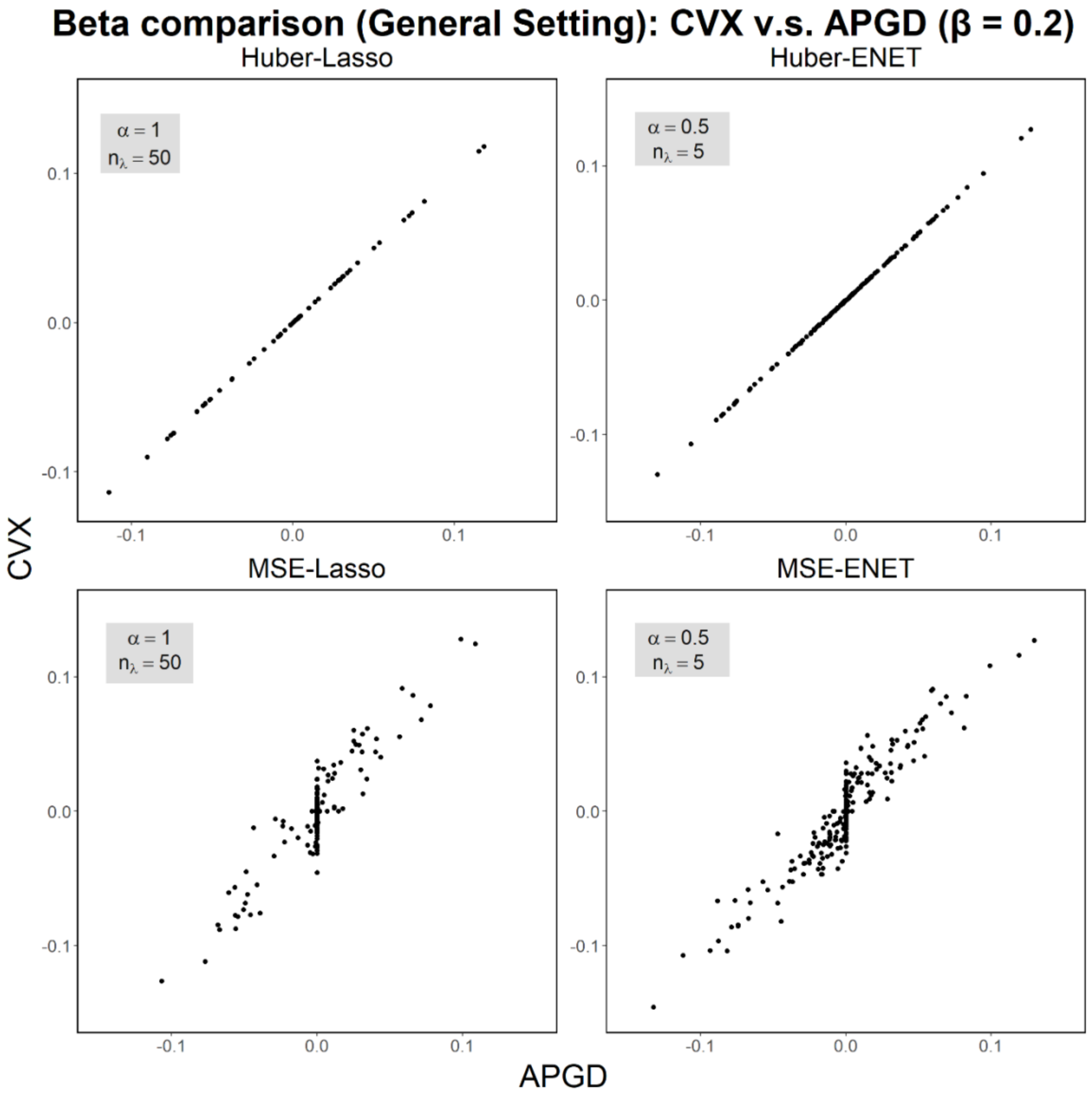

Figure S5. The estimation of regulation effects (beta) comparison of CVX versus APGD in the general setting ($\beta = 0.2$) by different algorithms.

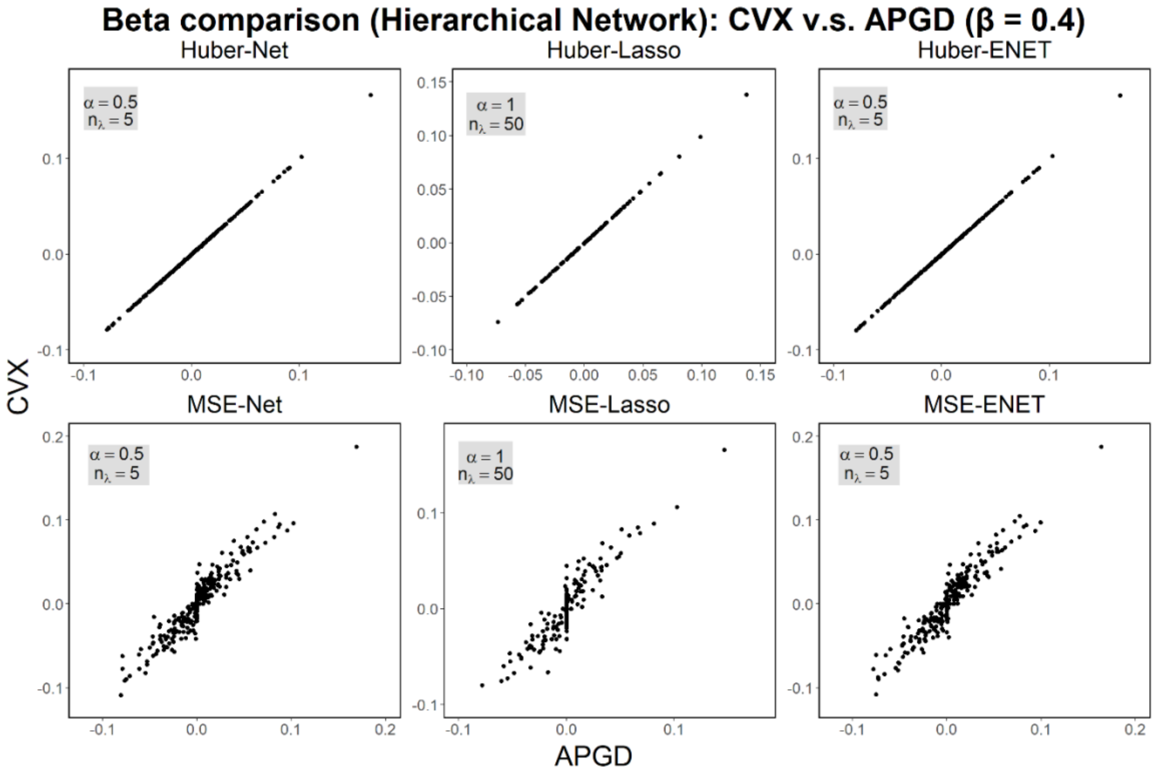

Figure S6. The estimation of regulation effects (beta) comparison of CVX versus APGD in the hierarchical network setting ($\beta = 0.4$) by different algorithms.

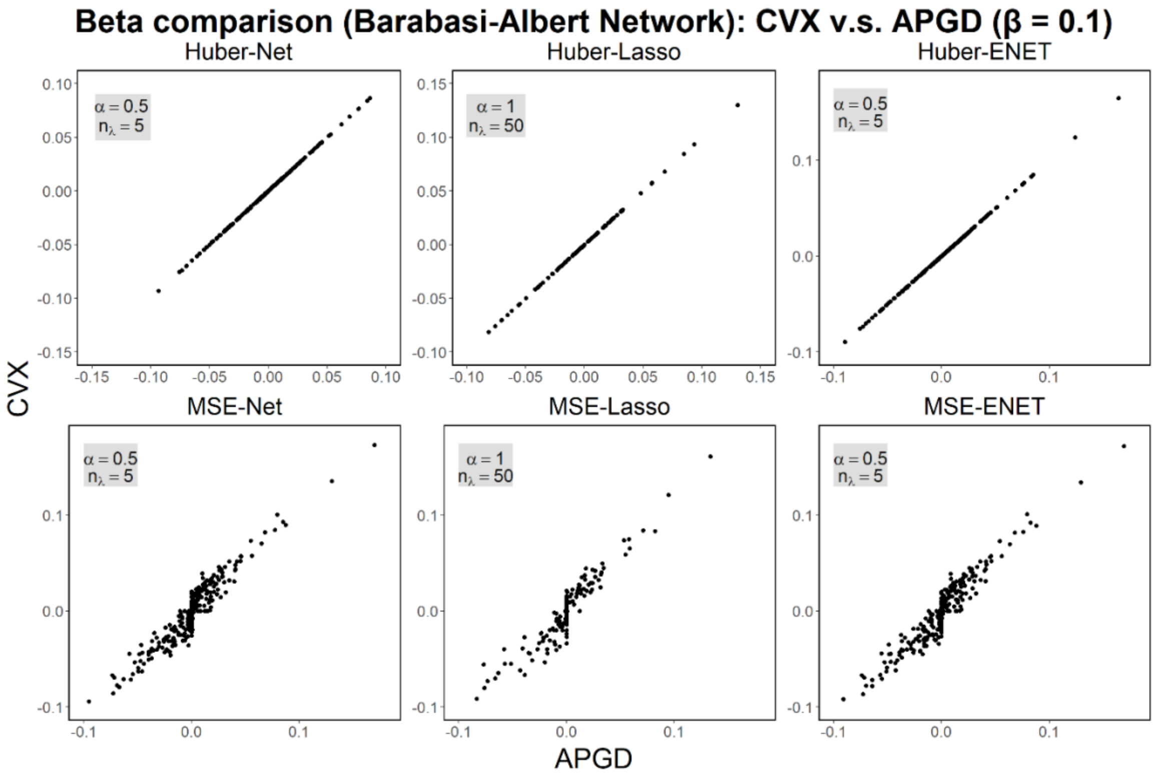

Figure S7. The estimation of regulation effects (beta) comparison of CVX versus APGD in the Barabasi-Albert network setting ($\beta = 0.1$) by different algorithms.

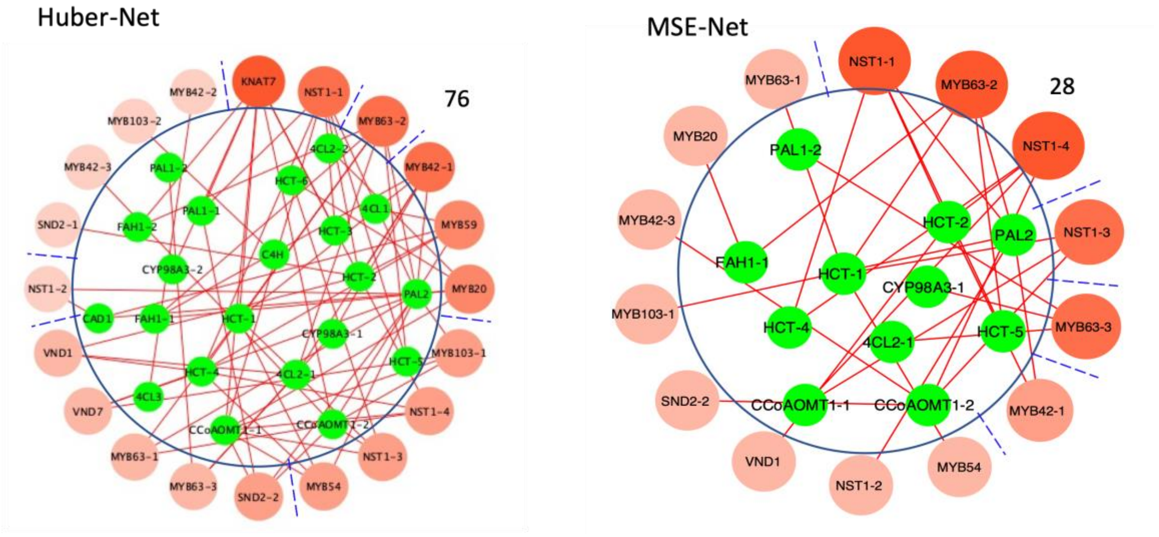

Figure S8. The gene regulatory networks of lignin pathway genes produced by the two Net methods, Huber-Net and MSE-Net. Each regulatory relationship has a selection probability equals to or larger than 0.9. The transcription factors are ranked in descending order by their connectivity to pathway genes in clockwise. The input data contained two subsets of transcriptomic data from maize B73**:** one was 2539 PWGs x 736 samples, and the other was 23 TFs x 736 samples.

Supplemental Texts

Text S1. The general simulation setting.

We considered the general simulation setting for comparison. To simulate expression levels of p target genes (TGs), we used the following linear model,

$$\boldsymbol{x}_i = y_i\boldsymbol{\beta} + \boldsymbol{\varepsilon}_i, \quad \text{(S7.1)}$$

Here $\boldsymbol{x}_i = (x_{i1}, \cdots, x_{ip})^T$ represents the expression level of p TGs in sample i. y_i is the expression level of a transcription factor (TF) in sample i and was generated from a standard normal distribution. $\boldsymbol{\beta} = (\beta_1, \cdots, \beta_p)^T$ represents the fixed regulation effects of the TF on p TGs. $\boldsymbol{\varepsilon}_i$ represents the error terms for p TGs in sample i, where $\boldsymbol{\varepsilon}_i$ was generated from a multivariate normal distribution with mean $\mathbf{0}$ and covariance matrix $\boldsymbol{I}_p$ (identity matrix), $\boldsymbol{\varepsilon}_i \sim MVN_p(\mathbf{0}, \boldsymbol{I}_p)$. We used $n = 300$ samples, $p = 500$ TGs in this simulation studies.

The regulation effects $\boldsymbol{\beta}$ were determined based on the relationship between TGs and the TF. In the general simulation settings, only the first 50 TGs were regulated by the TF. Therefore, the regulation effects $\boldsymbol{\beta}$ were defined as

$$\beta_j = \begin{cases} \beta, & if\ j \in (1, \cdots, 25), \\ -\beta, & if\ j \in (25, \cdots, 50), \\ 0, & otherwise. \end{cases} \quad \text{(S7.2)}$$

Text S2. Simulation settings if the target genes have the biological network structure.

To simulate correlated expression levels of p TGs within a biological network, we added the network factor into the general linear model,

$$\boldsymbol{x}_i = y_i\boldsymbol{\beta} + \boldsymbol{Z}_i + \boldsymbol{\varepsilon}_i, \qquad \text{(S8.1)}$$

Here $\boldsymbol{Z}_i \sim MVN_p(\mathbf{0}, \boldsymbol{\Sigma})$ is the network factor values in sample i with a network structure, where $\boldsymbol{\Sigma}$ is the covariance matrix of $\boldsymbol{x}_i$, and $\boldsymbol{Z}_i$ was generated from a multivariate normal distribution with mean $\mathbf{0}$ and covariance matrix $\boldsymbol{\Sigma}$. For a given network, $\boldsymbol{\Sigma}$ was simulated by the following ways, as described by Peng et al. (1) and Cao et al. (2). First, an initial concentration matrix is generated. For a pair of TGs m and k ($m = 1, \cdots, p, k = 1, \cdots, p$), the corresponding element in the initial concentration matrix was set as 0 if they were not linked or was generated from a uniform distribution on $[-0.7, -0.1] \cup [0.1, 0.7]$ if they were linked. Then the non-zero elements in the initial concentration matrix were rescaled to assure its positive definiteness and the rescaled matrix was averaged with its transpose to ensure the symmetry. Denote $W = (\omega_{mk})$ as the inverse of the matrix after rescaling and averaging based on the initial concentration matrix, the element Σ_{jk} in the covariance matrix $\boldsymbol{\Sigma}$ was determined by $\Sigma_{jk} = \omega_{mk}\sqrt{\omega_{mm}\omega_{kk}}$.

In this simulation, we used $n = 300$ samples and $p = 500$ TGs and considered two types of networks: hierarchical network and Barabasi-Albert network. For the hierarchical network, there were 5 disjointed subnetworks and each of them consisted of 100 TGs. The subnetwork was constructed as the same as Kim et al (3) (Figure S1). For Barabasi-Albert network, there were 50 subnetworks and each of them consisted of 10 TGs. For each subnetwork, a BA-based network was generated (4). For both types of networks, the network structure $\mathbf{A} = (a_{mk})$ of 500 TGs was constructed. $a_{mk} = 1$ if m^{th} TG and k^{th} TG were within the same subnetwork and $a_{mk} = 0$ otherwise.

The regulation effects $\boldsymbol{\beta}$ were determined based on the relationship between TGs and the TF. In the hierarchical network, only 45 TGs in the first subnetwork, which contained one centered TG and four groups of TGs denoted as g_1, g_2, g_3, and g_4, were regulated by the TF. Therefore, the regulation effects $\boldsymbol{\beta}$ were defined as

$$\beta_j = \begin{cases} \beta, & if\ TG\ j\ is\ the\ centered\ TG, \\ \beta/3 \times \sqrt{d_j}, & if\ j \in g_1\ or\ j \in g_3, \\ -\beta/3 \times \sqrt{d_j}, & if\ j \in g_2\ or\ j \in g_4, \\ 0, & otherwise. \end{cases} \quad \text{(S8.2)}$$

where d_j is the degree of TG j, which represents the number of TGs that were linked with TG j. In the Barabasi-Albert (BA)-based network, only 40 TGs in the first four subnetworks denoted as g_1, g_2, g_3, and g_4, were regulated by the TF. Therefore, the regulation effects $\boldsymbol{\beta}$ were defined as

$$\beta_j = \begin{cases} \beta \times \sqrt{d_j}, & if\ j \in g_1\ or\ j \in g_3, \\ -\beta \times \sqrt{d_j}, & if\ j \in g_2\ or\ j \in g_4, \\ 0, & otherwise. \end{cases} \quad \text{(S8.3)}$$

Text S3. APGD algorithm to solve Huber-Lasso.

In Huber-Lasso, we considered the Huber loss function and the Lasso penalty. Therefore, the penalized loss function can be decomposed as

$$f(\boldsymbol{\beta}) = g(\boldsymbol{\beta}) + h(\boldsymbol{\beta}) = \left(\sum_{i=1}^{n} H_M(y_i - \beta_0 - \boldsymbol{x}_i^T\boldsymbol{\beta})\right) + \left(\lambda\|\boldsymbol{\beta}\|_1\right). \tag{S1.1}$$

where $g(\boldsymbol{\beta})$ and $h(\boldsymbol{\beta})$ are given by

$$g(\boldsymbol{\beta}) = \sum_{i=1}^{n} H_M(y_i - \beta_0 - \boldsymbol{x}_i^T\boldsymbol{\beta}), \tag{S1.2}$$

$$h(\boldsymbol{\beta}) = \lambda\|\boldsymbol{\beta}\|_1. \tag{S1.3}$$

The APGD in k^{th} iteration can be defined as

$$\boldsymbol{\xi}^{k+1} := \boldsymbol{\beta}^k + \omega^k(\boldsymbol{\beta}^k - \boldsymbol{\beta}^{k-1})$$

$$\boldsymbol{\theta}^{k+1} := \boldsymbol{\xi}^{k+1} - \gamma^k \nabla g(\boldsymbol{\xi}^{k+1})$$

$$\boldsymbol{\beta}^{k+1} := \mathbf{Prox}_{\gamma^k h}(\boldsymbol{\theta}^{k+1}) \tag{S1.4}$$

where $\omega^k \in [0,1)$ is an extrapolation parameter and γ^k is the usual step size. These parameters must be chosen in specific ways to achieve convergence acceleration. One simple choice (5) for ω^k is $k/(k+3)$. Here $\nabla g(\boldsymbol{\xi}^{k+1})$ is the gradient of the convex differentiable function $g(\cdot)$ at $\boldsymbol{\xi}^{k+1}$, which can be calculated by

$$\nabla g(\boldsymbol{\xi}^{k+1}) = \sum_{i=1}^{n} -\nabla H_M(y_i - \beta_0 - \boldsymbol{x}_i^T\boldsymbol{\xi}^{k+1})\boldsymbol{x}_i. \tag{S1.5}$$

where let $\Delta_i := y_i - \beta_0 - \boldsymbol{x}_i^T\boldsymbol{\xi}^{k+1}$, then the gradient of Huber function can be calculated as $\nabla H_M(\Delta_i) = 2\Delta_i I(|\Delta_i| \leq M) + 2M sign(\Delta_i) I(|\Delta_i| > M)$. The operator $\mathbf{Prox}_{\gamma^k h}(\boldsymbol{\theta}^{k+1})$ is called proximal mapping for $h(\boldsymbol{\beta})$. To solve the Huber-Lasso, the key is to compute the proximal mapping for the convex non-differentiable function $h(\boldsymbol{\beta})$. It is not difficult to verify (6):

$$\mathbf{Prox}_{\gamma^k h}\left(\boldsymbol{\theta}^{k+1}\right) = \operatorname{argmin}_{\boldsymbol{\beta}}\left\{\lambda\|\boldsymbol{\beta}\|_1 + \frac{1}{2\gamma^k}\left\|\boldsymbol{\beta} - \boldsymbol{\theta}^{k+1}\right\|_2^2\right\}$$
$$= \operatorname{sign}\left(\boldsymbol{\theta}^{k+1}\right)\max\left\{\left\|\boldsymbol{\theta}^{k+1}\right\|_1 - \gamma^k\lambda, 0\right\}. \quad \text{(S1.6)}$$

To obtain a valid estimation in each iteration, we also defined an upper bound of $g(\cdot)$ as $\hat{g}_{\gamma^k}\left(\boldsymbol{\beta}, \boldsymbol{\xi}^{k+1}\right)$ which is given by

$$\hat{g}_{\gamma^k}\left(\boldsymbol{\beta}, \boldsymbol{\xi}^{k+1}\right) = g\left(\boldsymbol{\xi}^{k+1}\right) + \nabla g(\boldsymbol{\xi}^{k+1})^T\left(\boldsymbol{\beta} - \boldsymbol{\xi}^{k+1}\right) + \frac{1}{2\gamma^k}\left\|\boldsymbol{\beta} - \boldsymbol{\xi}^{k+1}\right\|_2^2. \quad \text{(S1.7)}$$

Algorithm S1 APGD for Huber-Lasso

function APGD.HuberLasso($\mathbf{X}, \boldsymbol{y}, \lambda$)
  Initiate $\boldsymbol{\beta}^0 = \boldsymbol{\beta}^1 = \mathbf{0}, \gamma = 1000$
  for $k \in 1 \ldots$ niter **do**
    $\boldsymbol{\xi}^{k+1} \leftarrow \boldsymbol{\beta}^k + k/(k+3) \times (\boldsymbol{\beta}^k - \boldsymbol{\beta}^{k-1})$
    Compute $\nabla g(\boldsymbol{\xi}^{k+1})$ from (S1.5)
    while TRUE **do**
      Compute $\boldsymbol{\beta}^{prox}$ from (S1.4)
      Compute $g(\boldsymbol{\beta}^{prox})$ from (S1.2) and $\hat{g}_{\gamma^k}(\boldsymbol{\beta}, \boldsymbol{\xi}^{k+1})$ from (S1.7)
      break if $g(\boldsymbol{\beta}^{prox}) \leq \hat{g}_{\gamma^k}(\boldsymbol{\beta}, \boldsymbol{\xi}^{k+1})$
      $\gamma \leftarrow \gamma \times 0.5$
    $\boldsymbol{\beta}^{k+1} \leftarrow \boldsymbol{\beta}^{prox}$
    break if $|f(\boldsymbol{\beta}^{prox}) - f(\boldsymbol{\beta}^k)| < 10^{-8}$ or $|\boldsymbol{\beta}^{prox} - \boldsymbol{\beta}^k| < 10^{-4}$
  return $\boldsymbol{\beta}^{prox}$

Text S4. APGD algorithm to solve Huber-ENET.

In Huber-ENET, we considered the Huber loss function and the Elastic Net penalty. Therefore, the convex differentiable function $g(\boldsymbol{\beta})$ and the convex non-differentiable function $h(\boldsymbol{\beta})$ are given by

$$g(\boldsymbol{\beta}) = \sum_{i=1}^{n} H_M(y_i - \beta_0 - \boldsymbol{x}_i^T\boldsymbol{\beta}) + \frac{1}{2}\lambda(1-\alpha)\boldsymbol{\beta}^T\boldsymbol{\beta},$$
$$h(\boldsymbol{\beta}) = \lambda\alpha\|\boldsymbol{\beta}\|_1. \tag{S2.1}$$

Therefore, the proximal operator in APGD for Huber-ENET and the gradient of convex differentiable function $g(\cdot)$ at $\boldsymbol{\xi}^{k+1}$, which can be calculated by the following formulas.

$$\mathbf{Prox}_{\gamma^k h}\left(\boldsymbol{\theta}^{k+1}\right) = \text{sign}\left(\boldsymbol{\theta}^{k+1}\right)\max\left\{\left\|\boldsymbol{\theta}^{k+1}\right\|_1 - \gamma^k\lambda\alpha, 0\right\} \tag{S2.2}$$

$$\nabla g(\boldsymbol{\xi}^{k+1}) = \sum_{i=1}^{n} -\nabla H_M\left(y_i - \beta_0 - \boldsymbol{x}_i^T\boldsymbol{\xi}^{k+1}\right)\boldsymbol{x}_i + \lambda(1-\alpha)\boldsymbol{\xi}^{k+1} \tag{S2.3}$$

Algorithm S2 APGD for Huber-ENET

function APGD.HuberENET($\mathbf{X}, \boldsymbol{y}, \lambda, \alpha$)
  Initiate $\boldsymbol{\beta}^0 = \boldsymbol{\beta}^1 = \mathbf{0}, \gamma = 1000$
  for $k \in 1 \ldots$ niter **do**
    $\boldsymbol{\xi}^{k+1} \leftarrow \boldsymbol{\beta}^k + k/(k+3) \times (\boldsymbol{\beta}^k - \boldsymbol{\beta}^{k-1})$
    Compute $\nabla g(\boldsymbol{\xi}^{k+1})$ from (S2.3)
    while TRUE **do**
      Compute $\boldsymbol{\beta}^{prox}$ from (S2.2)
      Compute $g(\boldsymbol{\beta}^{prox})$ from (S2.1) and $\hat{g}_{\gamma^k}(\boldsymbol{\beta}, \boldsymbol{\xi}^{k+1})$ from (S1.7)
      break if $g(\boldsymbol{\beta}^{prox}) \leq \hat{g}_{\gamma^k}(\boldsymbol{\beta}, \boldsymbol{\xi}^{k+1})$
      $\gamma \leftarrow \gamma \times 0.5$
    $\boldsymbol{\beta}^{k+1} \leftarrow \boldsymbol{\beta}^{prox}$
    break if $|f(\boldsymbol{\beta}^{prox}) - f(\boldsymbol{\beta}^k)| < 10^{-8}$ or $|\boldsymbol{\beta}^{prox} - \boldsymbol{\beta}^k| < 10^{-4}$
  return $\boldsymbol{\beta}^{prox}$

Text S5. APGD algorithm to solve Huber-Net.

In Huber-Net, we considered the Huber loss function and the network-based penalty. Therefore, the convex differentiable function $g(\boldsymbol{\beta})$ and the convex non-differentiable function $h(\boldsymbol{\beta})$ are given by

$$g(\boldsymbol{\beta}) = \sum_{i=1}^{n} H_M(y_i - \beta_0 - \boldsymbol{x}_i^T\boldsymbol{\beta}) + \frac{1}{2}\lambda(1-\alpha)\boldsymbol{\beta}^T\mathbf{S}^T\mathbf{LS}\boldsymbol{\beta}, \qquad h(\boldsymbol{\beta}) = \lambda\alpha\|\boldsymbol{\beta}\|_1. \tag{S3.1}$$

Therefore, the proximal operator in APGD for Huber-Net and the gradient of convex differentiable function $g(\cdot)$ at $\boldsymbol{\xi}^{k+1}$, which can be calculated by the following formulas.

$$\mathbf{Prox}_{\gamma^k h}\left(\boldsymbol{\theta}^{k+1}\right) = \mathrm{sign}\left(\boldsymbol{\theta}^{k+1}\right)\max\left\{\left\|\boldsymbol{\theta}^{k+1}\right\|_1 - \gamma^k\lambda\alpha, 0\right\} \tag{S3.2}$$

$$\nabla g(\boldsymbol{\xi}^{k+1}) = \sum_{i=1}^{n} -\nabla H_M\left(y_i - \beta_0 - \boldsymbol{x}_i^T\boldsymbol{\xi}^{k+1}\right)\boldsymbol{x}_i + \lambda(1-\alpha)\mathbf{S}^T\mathbf{LS}\boldsymbol{\xi}^{\boldsymbol{k+1}}. \tag{S3.3}$$

Algorithm S3 APGD for Huber-Net

function APGD.HUBERNET($\mathbf{X}, \boldsymbol{y}, \mathbf{L}, \mathbf{S}, \lambda, \alpha$)
  Initiate $\boldsymbol{\beta}^0 = \boldsymbol{\beta}^1 = \mathbf{0}, \gamma = 1000$
  for $k \in 1 \ldots$ niter **do**
    $\boldsymbol{\xi}^{k+1} \leftarrow \boldsymbol{\beta}^k + k/(k+3) \times (\boldsymbol{\beta}^k - \boldsymbol{\beta}^{k-1})$
    Compute $\nabla g(\boldsymbol{\xi}^{k+1})$ from (S3.3)
    while TRUE **do**
      Compute $\boldsymbol{\beta}^{prox}$ from (S3.2)
      Compute $g(\boldsymbol{\beta}^{prox})$ from (S3.1) and $\hat{g}_{\gamma^k}(\boldsymbol{\beta}, \boldsymbol{\xi}^{k+1})$ from (S1.7)
      break if $g(\boldsymbol{\beta}^{prox}) \leq \hat{g}_{\gamma^k}(\boldsymbol{\beta}, \boldsymbol{\xi}^{k+1})$
      $\gamma \leftarrow \gamma \times 0.5$
    $\boldsymbol{\beta}^{k+1} \leftarrow \boldsymbol{\beta}^{prox}$
    break if $|f(\boldsymbol{\beta}^{prox}) - f(\boldsymbol{\beta}^k)| < 10^{-8}$ or $|\boldsymbol{\beta}^{prox} - \boldsymbol{\beta}^k| < 10^{-4}$
  return $\boldsymbol{\beta}^{prox}$

Text S6. APGD algorithm to solve MSE-Lasso.

In MSE-Lasso, we considered the MSE loss function and the Lasso penalty. Therefore, the convex differentiable function $g(\boldsymbol{\beta})$ and the convex non-differentiable function $h(\boldsymbol{\beta})$ are given by

$$g(\boldsymbol{\beta}) = \frac{1}{2n}\sum_{i=1}^{n}(y_i - \beta_0 - \boldsymbol{x}_i^T\boldsymbol{\beta})^2, \qquad h(\boldsymbol{\beta}) = \lambda\|\boldsymbol{\beta}\|_1. \tag{S4.1}$$

Therefore, the proximal operator in APGD for MSE-Lasso and the gradient of convex differentiable function $g(\cdot)$ at $\boldsymbol{\xi}^{k+1}$, which can be calculated by the following formulas.

$$\mathbf{Prox}_{\gamma^k h}\left(\boldsymbol{\theta}^{k+1}\right) = \text{sign}\left(\boldsymbol{\theta}^{k+1}\right)\max\left\{\left\|\boldsymbol{\theta}^{k+1}\right\|_1 - \gamma^k\lambda, 0\right\} \tag{S4.2}$$

$$\nabla g(\boldsymbol{\xi}^{k+1}) = \frac{1}{n}\sum_{i=1}^{n} -\left(y_i - \beta_0 - \boldsymbol{x}_i^T\boldsymbol{\xi}^{k+1}\right)\boldsymbol{x}_i. \tag{S4.3}$$

Algorithm S4 APGD for MSE-Lasso

function APGD.MSELasso($\mathbf{X}, \boldsymbol{y}, \lambda$)
  Initiate $\boldsymbol{\beta}^0 = \boldsymbol{\beta}^1 = \mathbf{0}, \gamma = 1000$
  for $k \in 1 \ldots$ niter **do**
    $\boldsymbol{\xi}^{k+1} \leftarrow \boldsymbol{\beta}^k + k/(k+3) \times (\boldsymbol{\beta}^k - \boldsymbol{\beta}^{k-1})$
    Compute $\nabla g(\boldsymbol{\xi}^{k+1})$ from (S4.3)
    while TRUE **do**
      Compute $\boldsymbol{\beta}^{prox}$ from (S4.2)
      Compute $g(\boldsymbol{\beta}^{prox})$ from (S4.1) and $\hat{g}_{\gamma^k}(\boldsymbol{\beta}, \boldsymbol{\xi}^{k+1})$ from (S1.7)
      break if $g(\boldsymbol{\beta}^{prox}) \le \hat{g}_{\gamma^k}(\boldsymbol{\beta}, \boldsymbol{\xi}^{k+1})$
      $\gamma \leftarrow \gamma \times 0.5$
    $\boldsymbol{\beta}^{k+1} \leftarrow \boldsymbol{\beta}^{prox}$
    break if $|f(\boldsymbol{\beta}^{prox}) - f(\boldsymbol{\beta}^k)| < 10^{-8}$ or $|\boldsymbol{\beta}^{prox} - \boldsymbol{\beta}^k| < 10^{-4}$
  return $\boldsymbol{\beta}^{prox}$

Text S7. APGD algorithm to solve MSE-ENET.

In MSE-ENET, we considered the MSE loss function and the Elastic Net penalty. Therefore, the convex differentiable function $g(\boldsymbol{\beta})$ and the convex non-differentiable function $h(\boldsymbol{\beta})$ are given by

$$g(\boldsymbol{\beta}) = \frac{1}{2n}\sum_{i=1}^{n}(y_i - \beta_0 - \boldsymbol{x}_i^T\boldsymbol{\beta})^2 + \frac{1}{2}\lambda(1-\alpha)\boldsymbol{\beta}^T\boldsymbol{\beta},$$
$$h(\boldsymbol{\beta}) = \lambda\alpha\|\boldsymbol{\beta}\|_1. \tag{S5.1}$$

Therefore, the proximal operator in APGD for MSE-ENET and the gradient of convex differentiable function $g(\cdot)$ at $\boldsymbol{\xi}^{k+1}$, which can be calculated by the following formulas.

$$\mathbf{Prox}_{\gamma^k h}\left(\boldsymbol{\theta}^{k+1}\right) = \operatorname{sign}\left(\boldsymbol{\theta}^{k+1}\right)\max\left\{\left\|\boldsymbol{\theta}^{k+1}\right\|_1 - \gamma^k\lambda\alpha, 0\right\} \tag{S5.2}$$

$$\nabla g(\boldsymbol{\xi}^{k+1}) = \frac{1}{n}\sum_{i=1}^{n} -\left(y_i - \beta_0 - \boldsymbol{x}_i^T\boldsymbol{\xi}^{k+1}\right)\boldsymbol{x}_i + \lambda(1-\alpha)\boldsymbol{\xi}^{k+1} \tag{S5.3}$$

Algorithm S5 APGD for MSE-ENET

function APGD.MSEENET($\mathbf{X}, \boldsymbol{y}, \lambda, \alpha$)
  Initiate $\boldsymbol{\beta}^0 = \boldsymbol{\beta}^1 = \mathbf{0}, \gamma = 1000$
  for $k \in 1 \ldots$ niter **do**
    $\boldsymbol{\xi}^{k+1} \leftarrow \boldsymbol{\beta}^k + k/(k+3) \times (\boldsymbol{\beta}^k - \boldsymbol{\beta}^{k-1})$
    Compute $\nabla g(\boldsymbol{\xi}^{k+1})$ from (S5.3)
    while TRUE **do**
      Compute $\boldsymbol{\beta}^{prox}$ from (S5.2)
      Compute $g(\boldsymbol{\beta}^{prox})$ from (S5.1) and $\hat{g}_{\gamma^k}(\boldsymbol{\beta}, \boldsymbol{\xi}^{k+1})$ from (S1.7)
      break if $g(\boldsymbol{\beta}^{prox}) \le \hat{g}_{\gamma^k}(\boldsymbol{\beta}, \boldsymbol{\xi}^{k+1})$
      $\gamma \leftarrow \gamma \times 0.5$
    $\boldsymbol{\beta}^{k+1} \leftarrow \boldsymbol{\beta}^{prox}$
    break if $|f(\boldsymbol{\beta}^{prox}) - f(\boldsymbol{\beta}^k)| < 10^{-8}$ or $|\boldsymbol{\beta}^{prox} - \boldsymbol{\beta}^k| < 10^{-4}$
  return $\boldsymbol{\beta}^{prox}$

Text S8. APGD algorithm to solve MSE-Net.

In MSE-Net, we considered the MSE loss function and the network-based pnalty. Therefore, the convex differentiable function $g(\boldsymbol{\beta})$ and the convex non-differentiable function $h(\boldsymbol{\beta})$ are given by

$$g(\boldsymbol{\beta}) = \frac{1}{2n}\sum_{i=1}^{n}(y_i - \beta_0 - \boldsymbol{x}_i^T\boldsymbol{\beta})^2 + \frac{1}{2}\lambda(1-\alpha)\boldsymbol{\beta}^T\mathbf{S}^T\mathbf{L}\mathbf{S}\boldsymbol{\beta}, \qquad \text{(S6.1)}$$
$$h(\boldsymbol{\beta}) = \lambda\alpha\|\boldsymbol{\beta}\|_1.$$

Therefore, the proximal operator in APGD for Huber-Net and the gradient of convex differentiable function $g(\cdot)$ at $\boldsymbol{\xi}^{k+1}$, which can be calculated by the following formulas.

$$\mathbf{Prox}_{\gamma^k h}\left(\boldsymbol{\theta}^{k+1}\right) = \text{sign}\left(\boldsymbol{\theta}^{k+1}\right)\max\left\{\left\|\boldsymbol{\theta}^{k+1}\right\|_1 - \gamma^k\lambda\alpha, 0\right\} \qquad \text{(S6.2)}$$

$$\nabla g(\boldsymbol{\xi}^{k+1}) = \frac{1}{n}\sum_{i=1}^{n} -\left(y_i - \beta_0 - \boldsymbol{x}_i^T\boldsymbol{\xi}^{k+1}\right)\boldsymbol{x}_i + \lambda(1-\alpha)\mathbf{S}^T\mathbf{L}\mathbf{S}\boldsymbol{\xi}^{\boldsymbol{k+1}}. \qquad \text{(S6.3)}$$

Algorithm S6 APGD for MSE-Net

function APGD.MSENet($\mathbf{X}, \boldsymbol{y}, \mathbf{L}, \mathbf{S}, \lambda, \alpha$)
  Initiate $\boldsymbol{\beta}^0 = \boldsymbol{\beta}^1 = \mathbf{0}, \gamma = 1000$
  for $k \in 1 \ldots$ niter **do**
    $\boldsymbol{\xi}^{k+1} \leftarrow \boldsymbol{\beta}^k + k/(k+3) \times (\boldsymbol{\beta}^k - \boldsymbol{\beta}^{k-1})$
    Compute $\nabla g(\boldsymbol{\xi}^{k+1})$ from (S6.3)
    while TRUE **do**
      Compute $\boldsymbol{\beta}^{prox}$ from (S6.2)
      Compute $g(\boldsymbol{\beta}^{prox})$ from (S6.1) and $\hat{g}_{\gamma^k}(\boldsymbol{\beta}, \boldsymbol{\xi}^{k+1})$ from (S1.7)
      break if $g(\boldsymbol{\beta}^{prox}) \leq \hat{g}_{\gamma^k}(\boldsymbol{\beta}, \boldsymbol{\xi}^{k+1})$
      $\gamma \leftarrow \gamma \times 0.5$
    $\boldsymbol{\beta}^{k+1} \leftarrow \boldsymbol{\beta}^{prox}$
    break if $|f(\boldsymbol{\beta}^{prox}) - f(\boldsymbol{\beta}^k)| < 10^{-8}$ or $|\boldsymbol{\beta}^{prox} - \boldsymbol{\beta}^k| < 10^{-4}$
  return $\boldsymbol{\beta}^{prox}$

Supplemental Tables Legends

See Excel files attached separately

References

1. Peng, J., Wang, P., Zhou, N. and Zhu, J. (2009) Partial correlation estimation by joint sparse regression models. *Journal of the American Statistical Association*, **104**, 735-746.
2. Cao, X., Liang, X., Zhang, S. and Sha, Q. (2022) Gene selection by incorporating genetic networks into case-control association studies. *bioRxiv*.
3. Kim, K. and Sun, H. (2019) Incorporating genetic networks into case-control association studies with high-dimensional DNA methylation data. *BMC bioinformatics*, **20**, 1-15.
4. Albert, R. and Barabási, A.-L. (2002) Statistical mechanics of complex networks. *Reviews of modern physics*, **74**, 47.
5. Parikh, N. and Boyd, S. (2014) Proximal algorithms. *Foundations and Trends in optimization*, **1**, 127-239.
6. Polson, N.G., Scott, J.G. and Willard, B.T. (2015) Proximal algorithms in statistics and machine learning. *Statistical Science*, **30**, 559-581.
